# Molecular Thermal Motion Modulated Room-Temperature Phosphorescence for Multilevel Encryption

**DOI:** 10.34133/2022/9782713

**Published:** 2022-07-23

**Authors:** Jiaqiang Zhao, Guojuan Yan, Wei Wang, Shishi Shao, Binfang Yuan, Yan Jie Li, Xuepeng Zhang, Cheng Zhi Huang, Peng Fei Gao

**Affiliations:** ^1^Key Laboratory of Luminescence Analysis and Molecular Sensing (Southwest University), Ministry of Education, College of Pharmaceutical Sciences, Southwest University, Chongqing 400715, China; ^2^Chongqing Key Laboratory of Inorganic Special Functional Materials, College of Chemistry and Chemical Engineering, Yangtze Normal University, Fuling, Chongqing 408100, China; ^3^Hefei National Laboratory for Physical Science at the Microscale, University of Science and Technology of China, 96 Jinzhai Rd, Hefei, Anhui 230026, China

## Abstract

The stimulus-responsive room-temperature phosphorescence (RTP) materials have become an increasingly significant topic in the fields of bioimaging, sensing, and anticounterfeiting. However, this kind of materials is scarce to date, especially for the ones with delicate stimulus-responsive behavior. Herein, a universal strategy for multilevel thermal erasure of RTP via chromatographic separation of host-guest doping RTP systems is proposed. The tunable host-guest systems, matrix materials, heating temperature, and time are demonstrated to allow precise six-level data encryption, QR code encryption, and thermochromic phosphorescence encryption. Mechanistic study reveals that the thermal-responsive property might be attributed to molecular thermal motion and the separation effect of the silica gel, which provides expanded applications of host-guest RTP materials such as cold chain break detection. This work offers a simple yet universal way to construct advanced responsive RTP materials.

## 1. Introduction

Purely organic room-temperature phosphorescence (RTP) materials with long lifetime have been widely applied in various areas, such as organic optoelectronics, biomedicine, optical sensing, and data encryption [1, 2]. Over the past decade, strategies including halogen bonding [3, 4], charge-transfer mediation [5–7], molecular-orbital hybridization [8, 9], crystal engineering [10–14], polymer-matrix assistance [15–20], and host-guest doping [21–26] have been utilized to boost intersystem crossing and diminish nonradiative decays to realize organic RTP. Accordingly, ultralong RTP systems with emission wavelength even at near-infrared region can be achieved [27–31], which is promising in background-free bioimaging. However, for imaging analysis, sensing, and information encryption, RTP materials that could respond and exhibit luminescence changes to external stimulus are more expected. But such kind of materials are less developed, especially for the ones with sophisticated stimulus-responsive behaviors [32, 33].

RTP materials that can respond to a specific stimulus (such as illumination [34, 35], mechanical force [36, 37], acidity/alkalinity [38], and water treatment [39, 40]) have been reported with very limited cases. Among these stimulus-responsive RTP systems, both single-component and multicomponent systems were investigated [33]. The stimulus-responsive behavior of the single-component systems can result from the changes in molecular structure or packing mode of the RTP molecule upon exposure to a stimulus. On the other hand, multicomponent stimulus-responsive ones, such as host-guest doping systems, are sensitive to the distance and orientation between the each component which can be tuned by heating and grinding [37, 41]. However, the reported stimulus-responsive behaviors of host-guest system to date mainly focused on reduction of the distance between the host-guest components to turn on the RTP.

Another neglected aspect is to increase the distance between the components, which seems to be rather simple and can be facilely achieved by separating each species. This approach should be as important as the stimulus-triggered decrease of the distance between involved dyes, just like the equally useful construction (turn on) and prevention (turn off) of the distance-sensitive Förster resonance energy transfer (FRET) [42]. Obviously, the separation efficiency is of vital significance, which is mainly determined by two processes, namely, the separation of the host and guest components and the prevention of their combination. However, up to now, these two processes are difficult to be achieved, especially at the molecular level. Recently, extralow level of guest has been confirmed to be able to achieve bright RTP [24, 43], which leads to greater separation difficulty of the host-guest systems. And thus, the separation-based strategy for controllable regulation of RTP emission remains a formidable challenge.

**Figure 1:**
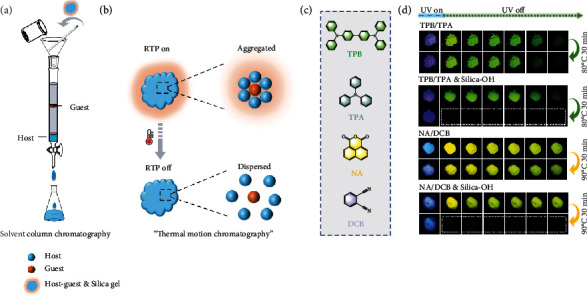
The silica gel induced thermal-responsive host-guest RTP systems. (a) Conventional solvent column chromatographic separation. (b) “Heating-promoted chromatographic separation based on molecular thermal motion”. (c) The molecular structures of TPB, TPA, NA, and DCB molecules. (d) The phosphorescence images of the TPB/TPA, TPB/TPA & silica-OH, NA/DCB, and NA/DCB & silica-OH before and after heating treatment. TPB/TPA systems: 80°C, NA/DCB systems: 90°C.

Inspired by the excellent separation ability of the universal column chromatography, herein, a universal chromatographic separation method is successfully developed for the thermal erasure (turn-off) of the RTP emission in host-guest systems for the first time (Figures 1(a) and 1(b)). The ubiquitous molecular thermal motion is proved to be the basis for the thermally accelerated separation process, and the combination of the separated host-guest components after cooling could be prevented by the most widely used chromatographic separation matrix, silica gel. The normal-phase silica gel with higher affinity to molecules exhibits higher quenching efficiency and lower response temperature for the same RTP systems than the reversed-phase silica gel does. To demonstrate the generality of this strategy, their different temperature-response properties greatly enrich the dimension of encryption and show the potential application in cold chain break detection. In addition to the erasure of the RTP, thermochromic phosphorescence encryption is easily realized via the combination of the colorful host-guest doping systems. Therefore, this cost-effective and multilevel encryption strategy would be used for constructing the next-generation anticounterfeiting and quality control technology.

## 2. Results

### 2.1. Thermal Responsive RTP Materials

Two green (TPB/TPA and 2,3-NA/PCP, abbreviations: tetraphenylbenzidine for TPB, triphenylamine for TPA, 2,3-naphthalenedicarboxylic anhydride for 2,3-NA, and 2,3,4,5,6-pentachlorpyridine for PCP) [21, 44] and two yellow (NA/DCB and NA/ABDO, abbreviations: 1,8-naphthalic anhydride for NA, 1,2-dicyanobenzene for DCB, and 6-acetyl-1,4-benzodioxane for ABDO) [21] host-guest RTP systems were used as models to investigate the following thermal-responsive RTP design (Figure 1(c)). The construction of the RTP systems was simply achieved through melt-casting method [5, 21], and they showed similar RTP properties as in the references, including emission bands and lifetimes. Before the stimulus-responsive exploration, the thermal stability of these RTP systems was firstly tested. After the heating and cooling operations, there were imperceptible emission differences from the initial status, demonstrating that direct heating treatment could not lead to separation of host-guest components and RTP emission response. Once combined with normal-phase silica gel (abbreviated as silica-OH), the most commonly used column chromatography separation matrix, the heating triggered quenching of RTP emission in all of these four RTP systems and three other host-guest doping RTP systems, including TPB/TPP, NA/TMBN, and NA/PCP (abbreviations: triphenylphosphine for TPP and 3,4,5-trimethoxybenzonitrile for TMBN) (Figure 1(d) and Fig. S1-S2). As the most widely used column packing material of high-performance liquid chromatography (HPLC), reversed-phase silica gel (abbreviated as silica-OR) could lead to a noticeable but weaker RTP emission moderating effect than silica-OH did (Fig. S3).

To evaluate the contributions of the chromatographic separation matrix, another column chromatographic separation matrix, neutral alumina, and some other solid matrix (including sodium chloride (NaCl), potassium chloride (KCl), sodium sulfate (Na_2_SO_4_), refined white sugar, flour, and starch) were used to investigate their performance in regulating the thermal-responsive property of these RTP systems. As expected, their regulating performance was deeply dependent on the separation performance, which was mainly determined by the fluffiness and affinity. Flour and starch could exhibit weak contributions to this regulation, but neutral alumina showed acceptable regulatory capacity, similar to silica-OR (Fig. S4). Based on these results, silica-OH and silica-OR, which had high-quality specification and wide applicability, were used for the following studies.

During column chromatography, a reasonable silica gel-to-sample mass ratio is a prerequisite for effective sample separation. For silica-OH, the rich optional mesh sizes are also important factors to tune the separation performance. If the chromatographic separation is the working principle of this thermal-responsive design, these two parameters should exhibit considerable regulation performance. Three silica gel-to-sample mass ratios (1 : 1, 1 : 5, and 1 : 10) for both silica-OH and silica-OR and three mesh sizes (60-100, 300-400, and 500-800 mesh) for silica-OH were used to verify this hypothesis. The results showed that with the increase of the silica gel to sample mass ratio, higher performance of the thermal erasure of the RTP could be obtained (Fig. S5-S6). Notably, although resulting in higher sensitivity, excessive silica gel to sample ratio would also lead to a weak initial RTP emission intensity. It was also as expected that the silica-OH with larger mesh exhibited higher RTP quenching efficiency (Fig. S7). The silica gel-to-sample mass ratio 1 : 5 and the 300-400 mesh silica-OH were selected for the further investigations.

**Figure 2:**
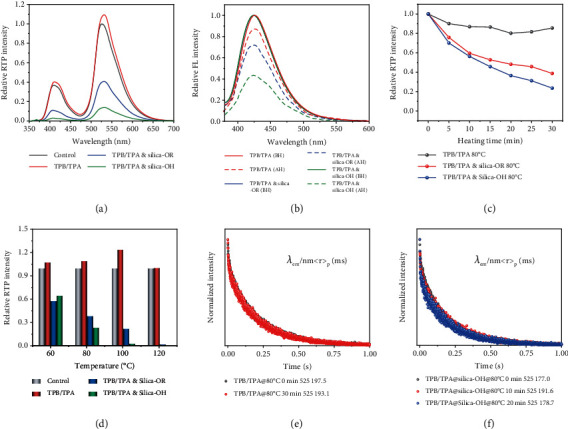
Spectra and phosphorescence lifetime characteristics of the thermal response of TPB/TPA and TPB/TPA & silica gel. (a) The relative phosphorescence spectra of the TPB/TPA, TPB/TPA & silica-OR, and TPB/TPA & silica-OH before and after thermal treatment (80°C 30 min). (b) The relative fluorescence spectra and (c) phosphorescence intensity trends of the groups in (a). (d) The relative phosphorescence intensity of TPB/TPA, TPB/TPA & silica-OR, and TPB/TPA & silica-OH after thermal treatment (60, 80, 100, and 120°C) for 30 min. (e) The phosphorescence lifetime of TPB/TPA before and after heating at 80°C 30 min. (f) The phosphorescence lifetime of TPB/TPA & silica-OH before and after heating at 80°C 10 min and 20 min. The control in (a) means the initial phosphorescence intensity of the each group was normalized to “1.” Abbreviations: BH: before heating; AH: after heating. In (e) and (f), *λ*_ex_ = 365 nm.

The thermal-responsive property of all these four RTP systems was systematically investigated at a range of temperature from room temperature to about ten degrees below the melting point of the host molecules. Overall, all of the selected RTP systems showed temperature-dependent thermal-responsive property in the silica gel matrix, and the silica-OH always led to a higher sensitivity (Figure 2(a) and Fig. S8-S12). The NA/ABDO systems exhibited the highest sensitivity in both silica-OH and silica-OR, while NA/DCB showed excellent stability in silica-OR. The different thermal response sensitivity was useful for multilevel stimulus-responsive applications. The fluorescence spectra of these RTP systems in silica-OH and silica-OR exhibited low correlation with the thermal treatment at a certain temperature (Figure 2(b) and Fig. S13-S16), which should be attributed to the single molecular emission was free from the regulation of chromatographic separation. This results also demonstrated that the two-component doping systems were the prerequisite of this thermal-responsive strategy.

By comparing the variation trend of phosphorescence intensities in 30 minutes at a certain temperature and the trend of phosphorescence intensities at the 30 min at various temperatures, it could be concluded that the response was much sensitive to the environmental temperature, instead of the cumulative time at a certain temperature (Figures 2(c) and 2(d) and Fig. S17). In such case, a long time of a lower temperature treatment could be used as a low-level decryption operation, which had little effect on the subsequent high-level decryption operations. Obviously, this was important for the design of decryption operation for multilevel data encryption applications. Based on the above investigation of temperature-dependent thermal response, the temperature close to the melting point of the host molecules turned to be an effective trigger to erase the RTP emission, demonstrating that the molecular thermal motion played important roles in this process. After the heating and cooling operation, the RTP lifetime of the host-guest systems showed no obvious changes (Figure 2(e) and Fig. S18a), indicating the thermal treatment could not lead to the separation of the components. Besides, during the thermal erasure of the RTP, the lifetime of the TPB/TPA & silica-OH and NA/DCB & silica-OH systems also maintained the initial level (Figure 2(f) and Fig. S18b), suggesting that the gradually reduced RTP emission could be attributed the gradually increased separated host-guest proportions.

### 2.2. Mechanism of the Thermal Responsive Property

**Figure 3:**
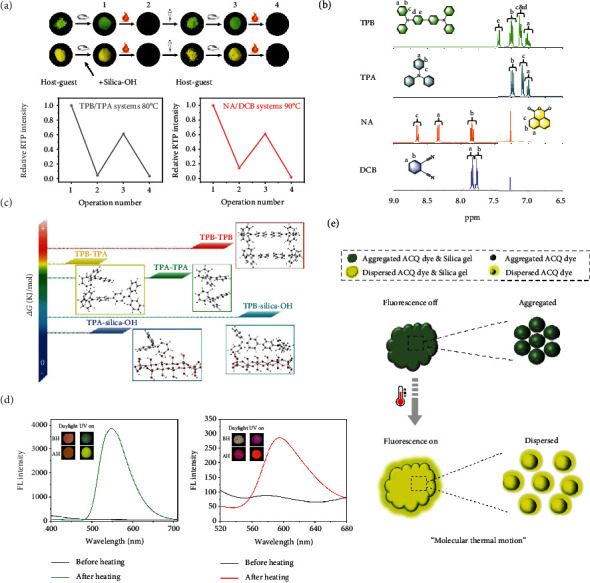
Experimental and theoretical investigation of the mechanism. (a) Recoverability measurement of the thermal-erased RTP of NA/DCB & silica-OH and TPB/TPA & silica-OH systems (top). Photos of phosphorescence emission of host-guest and host-guest & silica-OH (down). The corresponding relative phosphorescence intensities of host-guest & silica-OH during heating and recovering. Water was added during the extraction process. (b) ^1^H NMR spectra of the TPB, TPA, NA, and DCB molecules extracted from the thermal erased TPB/TPA & silica-OH and NA/DCB & silica-OH systems (dissolve in deuterated chloroform). (c) Gibbs free energy change trends of the two-components in TPB/TPA & silica-OH systems (298.15 K). (d) Photographs and fluorescence spectra of the thermal triggered molecular diffusion of fluorescein (230°C 30 min, left) and rhodamine B (180°C 30 min, right). (e) Diagram of the thermal accelerated diffusion.

To determine the mechanism of this strategy, the recoverability of the thermal-erased RTP was explored. After the thermal erasure of the RTP of NA/DCB & silica-OH, dichloromethane (CH_2_Cl_2_) was firstly added to extract the NA and DCB molecules from the silica-OH, then the RTP emission of this hybrid system after a drying and grinding treatment was measured. The recovery efficiency of the RTP intensity was about 27.18%, and the recovery efficiency could maintain at this level during five more repeating cycles, demonstrating that this RTP emission was able to be restored partially (Fig. S19). The limited recovery efficiency should be attributed to the inherent elution efficiency of this method. To further increase the recovery efficiency, during the elution process, water was added to achieve a better extraction effect. As known, the silica-OH was easily dissolved in water, which could be helpful for the extraction of the molecules from the silica-OH into the CH_2_Cl_2_. As a result, a much higher recovery efficiency of the RTP emission for both NA/DCB (61.27%) and TPB/TPA (61.29%) systems was achieved (Figure 3(a)), and this phenomenon further confirmed that the thermal-responsive property was more likely to rely on a physical process.

In order to explore the molecular structure information of the host and guest components during the thermal erasure of RTP emission, high-resolution mass spectrometry (HRMS) and ^1^H nuclear magnetic resonance (NMR) spectra were used to characterize the molecular structure after the thermal-responsive behavior. As the extraction of the guest molecule from the doping system was difficult due to low guest concentration, a suitable host-guest ratio (1 : 50) which took into account both the RTP emission and extraction effect was selected for the following structure determination. Firstly, from the HRMS results, both the host-guest molecules could be determined from the extraction of the TPB/TPA & silica-OH (TPB^+^ 488.2249; [TPA+H]^+^ 246.1269) and the NA/DCB & silica-OH systems ([NA+H]^+^ 199.0382; [DCB+H]^+^ 129.0444), which was consistent with the HRMS date of the directly doped TPB/TPA (TPB^+^ 488.2250; [TPA+H]^+^ 246.1270) and NA/DCB systems ([NA+H]^+^ 199.0381; [DCB+H]^+^ 129.0447) (Fig. S20-S21). Only two components were extracted from both the TPB/TPA & silica-OH and the NA/DCB & silica-OH systems after the stimulus-responsive behavior, and the ^1^H NMR spectrum characteristics revealed that the extracted molecules were still the initial host-guest molecules (Figure 3(b)). At this stage, it was confirmed that there were only physical interaction processes during the thermal erasure of the RTP emission.

Fourier transform infrared (FTIR) spectroscopy and X-ray diffraction (XRD) analysis were used to investigate the interactions between the host-guest RTP systems and silica gel during the thermal erasure of RTP emission. The FTIR signal was weak because of the low content of the RTP systems in the complex (including the silica gel and potassium bromide matrix) and showed no obvious changes from the weak signal (Fig. S22). An obviously decreased intensities in the peaks could be observed from the XRD results after the thermal treatment, especially for the silica-OH groups. This indicated the affinity between the host-guest systems and the silica-OH indeed lead to the changes in the physical state and the related RTP emission. A much weak change in the peak intensity was observed for the silica-OR, especially for the NA/DCB systems. This result was consistent with the lower quenching efficiency of silica-OR (Fig. S23-S24). As a result, for most of the used host and guest molecules with strong polarity, the silica-OH with higher affinity and chromatographic separation capability always exhibit excellent regulation efficiency in the RTP emission.

To get a deeper insight into the thermal-triggered separation mechanism of this strategy, the intermolecular affinities between the host-host, host-guest, and guest-guest and the affinities between the host-silica gel and guest-silica gel, at room temperature and about ten degrees below melting point, were obtained by the Gaussian simulation. The periodically distributed silanols were used as the model structure of silica-OH[45], and due to the complexity of the separation process of silica-OR, such as the polyalkane chain synergy and the multiple interactions [46, 47], the silica-OR was no longer simulated herein. Gibbs free energy change (*∆G*) was used to describe the affinity and the possible trends of the molecular separation and adsorption (Figure 3(c) and Tables 1 and 2). Firstly, the *∆G* values showed that at higher temperature, the affinities between all the two-component combinations decreased, which facilitated the separation of the initially fused host-guest systems. Secondly, the *∆G* values showed that no matter at room temperature or high temperature, both the host and guest molecules preferred to bind to silica-OH rather than the small molecules themselves, which was beneficial for preventing the recombination of host-guest systems. Overall, these results were consistent with the experimental data and provided a quantitative microscopic understanding of the possible thermal-triggered separation mechanism.

The widely used host molecules in the host-guest doping RTP systems were always simple molecules with negligible absorption and fluorescence in the visible region, such as the TPA and DCB, which were difficult to be used to confirm the molecular thermal motion and diffusion process during the thermal erasure of RTP emission. Herein, two classical fluorescent molecules, fluorescein and rhodamine B, with aggregation caused quenching (ACQ) characteristics were used to display the thermal increased molecular motion and diffusion on to the surface of the silica-OH. In contrast to aggregation-induced emission (AIE) phenomenon [48], fluorescein and rhodamine B showed weak fluorescence at the initial aggregate state mixed with silica-OH (1 : 40) but showed enhanced fluorescence intensity after the thermal treatment (Figure 3(d)), and this fluorescence enhancement could also be discovered at room temperature for a long period of time. These results revealed the thermal accelerated diffusion of the molecules on to the surface of the silica-OH (Figure 3(e)), which should also apply to the host-guest & silica-OH systems. Compared to the silica-OH, no obvious thermal accelerated diffusion of fluorescein and rhodamine B in the NaCl matrix was determined from the fluorescence intensity changes (Fig. S25). In addition, for NaCl, without separation ability, the combination of the separated host-guest systems could not be prevented efficiently (Fig. S26), demonstrating the chromatographic separation effect of silica gel was also indispensable. The aforementioned experimental and theoretical explorations consistently suggested the possible mechanism of this thermal erasure strategy of RTP could be attributed to the thermally promoted molecule movement and the chromatographic separation effect of the silica gel (Fig. S27). As an important parameter of this stimulus-responsive materials, the responsive temperatures could be designed based on the possible influencing factors, including the melting point of the host molecule, the intermolecular forces, the silica gel matrix, and the ratio between the host-guest RTP systems and the matrix.

### 2.3. Thermal Responsive RTP for Multilevel Information Encryption and Cold Chain Break Detection

Based on this strategy, a multilevel information encryption application was developed. Firstly, the available single stimulus, which might be used as a layer of information encryption of the multilevel information encryption application, was systematically investigated (“butterflies” pattern with yellow RTP emission and “leaves” pattern with green RTP emission, Fig. S28). The switch between the RTP and fluorescence induced by the UV irradiation on and off could be as the simplest stimulus. The thermal erasure of the RTP emission of the NA/DCB & silica-OH and TPB/TPA & silica-OH systems could be regarded as a single stimulus (90°C 30 min for NA/DCB, 80°C 30 min for TPB/TPA). The RTP systems with high response temperature, which was mainly determined by the melting point of the host molecules, could provide unlimited design possibilities. This design space was important for the multilevel information encryption (Fig. S29). For NA/DCB & silica-OH and TPB/TPA & silica-OH systems, their emission maintained under a mild thermal treatment (60°C 20 min) but was turned off clearly under a severe thermal treatment (120°C 5 min). Obviously, the mild thermal treatment could be regarded as an effective stimulus for the erasure of the NA/ABDO & silica-OH and TPB/TPP & silica-OH systems. Thanks to the optional host-guest systems, the two yellow emissive NA/ABDO & silica-OH and NA/DCB & silica-OH systems could be sequentially quenched (70°C 5 min for NA/ABDO). Similarly, a mild thermal treatment (70°C 20 min) could selectively erase the RTP emission of TPB/TPP & silica-OH. Among the solid matrix without separation ability, the cost-effective NaCl was ideal to be used for constructing the nonresponsive RTP systems. In such case, although the RTP emission of the NA/DCB & silica-OH and TPB/TPA & silica-OH systems could be erased easily at the first level thermal treatment (90°C 30 min for NA/DCB and 80°C 30 min for TPB/TPA), both the RTP emission of these two systems in NaCl were particularly stable to a severe thermal treatment (120°C 30 min). Some other treating conditions could also be designed, for example, the silica-OR would lead to a higher response temperature than silica-OH. Moreover, by adjusting the mass ratio between the host-guest systems and the silica gel, the response sensitivity as well as the RTP emission intensity could also be effectively regulated, which could serve as a special encryption parameter for advanced anticounterfeiting.

**Figure 4:**
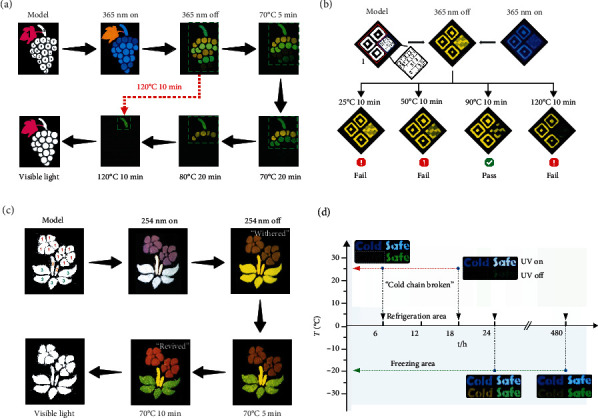
Multilevel thermal-responsive RTP materials for information encryption and detection of cold chain break. (a) Six-level information encryption application of the thermal-responsive RTP systems (component 1 for rhodamine B & silica-OH, 2 for TPB & silica-OH, 3 for NA/ABDO & silica-OH (1 : 10), 4 for TPB/TPP & silica-OH (1 : 10), 5 for NA/ABDO & silica-OH (1 : 1), 6 for TPB/TPP & silica-OR (1 : 3), 7 for NA/DCB & silica-OH (1 : 1), and 8 for TPB/TPP & NaCl (1 : 5)). (b) Information encryption “QR code” constructed by NA/DCB system and three different matrix (component 1 for NA/DCB & NaCl (1 : 5), 2 for NA/DCB & silica-OR (1 : 5), and 3 for NA/DCB & silica-OH (1 : 5). (c) The thermochromic RTP system for information encryption (component 1 for 2,3-NA/PCP & silica-OH (1 : 5)+Py/PCP & NaCl (1 : 5), 2 for NA/PCP & NaCl (1 : 20), and 3 for NA/PCP & silica-OH (1 : 5) +2,3-NA/PCP & NaCl). (d) Monitoring of two kinds of simulated cold chain break with two yellow and green RTP systems (“cold” for NA/ABDO & silica-OH (1 : 10) and “safe” for TPB/TPP & silica-OH (1 : 10)).

By combining these optional single stimulus elements, a multilevel information encryption in a “Grape” pattern was designed (Figure 4(a)). Under UV irradiation, orange colored component 1 and blue colored component 2 to 8 could be observed. After the UV irradiation ceased, yellow colored components 3, 5, and 7 and green colored components 4, 6, and 8 were left. The fluorescent components 1 and 2 disappeared, and the component 3 showed weak RTP emission due to the low mass ratio of NA/ABDO. After the first thermal treatment (70°C 5 min), the RTP emission of component 3 disappeared and the components 4 and 5 turned weaker. The second thermal treatment (70°C 20 min) led to the quenching of the RTP emission of components 4 and 5. After the third thermal treatment (80°C 20 min), the component 6 disappeared. Then the fourth thermal treatment (120°C 10 min) led to the quenching of the component 7. Up to now, all the grapes disappeared. In fact, this result could also be directly achieved by the fourth thermal treatment, in such case, only a third-level information encryption could be realized. However, the fluorescence and the gradual thermal erasure of the RTP emission achieved an advanced six-level information encryption.

Based on a simple combination of the NA/DCB in three matrix (NaCl, silica-OR, and silica-OH), a precise QR code encryption could be realized (Figure 4(b)). Both the first (25°C 10 min) and second (50°C 10 min) thermal treatment, which was too mild to erase the RTP of NA/DCB & silica-OR, could not lead to the right QR code information. And the severe fourth condition (120°C 10 min) induced the erasure of both the RTP of NA/DCB & silica-OR and NA/DCB & silica-OH, which also missed the correct information. Obviously, the limited treatment condition endows the QR code with a higher level of anticounterfeiting capability, which could be regarded as advanced anticounterfeiting technology [49].

In addition to the RTP erasure based information encryption, this thermal responsive RTP strategy was used to construct thermochromic RTP materials, which could further enrich the information encryption applications. A new red emissive host-guest RTP system (Py/PCP, abbreviations: pyrene for Py and pentachloropyridine for PCP) was developed and characterized (Fig. S30). After the heating for 30 minutes at 70°C, the red RTP emission could be erased (Fig. S31). Then, this red emissive system, a yellow emissive NA/PCP system, and a green emissive 2,3-NA/PCP system were used to construct thermochromic RTP materials (Figure 4(c)). After the thermal treatment (70°C 10 min), the brown colored “flower” turned to red color due to the stable red RTP and the thermal responsive green RTP. The “leaf” changed from the initial yellow color into the green color, and the “stem” maintained the yellow color. Overall, this “withered” plant gradually rejuvenated, demonstrating an artistic multilevel encryption.

The utilization of this strategy based on stimulus-responsive RTP materials was further investigated. The molecular thermal motion was confirmed to be the fundamental driving force of the thermal erasure of the RTP emission, and thus, the response of some systems could be triggered by relatively low temperatures such as room temperature (25°C). According to the proposed mechanism, these sensitive systems might be still able to be stable at lower temperatures, such as the classic refrigeration temperature (0 to 4°C) and the freezing temperature (-20°C). In contrast to the traditional phosphors exhibiting dramatic differences for ultralow (liquid nitrogen temperature) and room temperature, herein, these systems could be used for determination or monitoring temperature changes at this narrow range (-20 to 25°C), which would be much more useful for the daily life applications. In such case, a model application, the cold chain break detection, was explored. As well known, to ensure the safety and effectiveness of the food, medicine, biochips, and some biodiagnostic reagents, there are always need for refrigerated storage and cold chain transportation [50]. The failure of the cold chain always leads to health threat and economic losses. To identify failures of cold chain, cost-effective, sensitive, and reliable temperature sensors are urgently needed [51, 52].

The NA/ABDO & silica-OH (1 : 10, in the pattern of “cold”) and TPB/TPP & silica-OH (1 : 10, in the pattern of “safe”) systems, with different sensitivities in this temperature range, were selected to monitor the simulated break of the cold chain (Figure 4(d) and Fig. S32). The RTP emission of these two probes was stable within 24 hours at -20°C (simulated normal cold chain). In fact, on day 20, the green RTP was still bright and the yellow RTP could also be identified, suggesting that the molecular motion still existed at this temperature. However, once experienced a 6 hour of 25°C (simulated failed cold chain), the yellow emission of the “cold” pattern was quenched thoroughly. At this moment, the green emission of the “safe” pattern was still kept, demonstrating the products was pending and needed to be further analyzed. Both of these two RTP systems were quenched after the experience of 18 hours at 25°C, suggesting the complete failure of the cold chain. This simple exploration demonstrated the potential applications of this thermal responsive RTP systems in the field of quality control in refrigerated storage and cold chain transportation.

## 3. Discussion

In conclusion, a general strategy to construct thermal-responsive RTP materials was developed. Thanks to the flourishing host-guest doping RTP materials, tunable colors can be introduced. Combined with the plentiful choice of the silica gel and RTP systems with diffident melting points, a wide range of response temperatures could be obtained easily for multilevel information encryption. Because an extralow level of guest is able to achieve bright RTP, this thermal-responsive RTP materials might be used for the analysis of trace substances, for example, the trace impurity analysis in pharmaceutical analysis. Investigations into the mechanism demonstrate that both the molecular thermal motion and the separation function of the silica gel contributed significantly to increase the distance between the components of host-guest RTP systems. In addition to the two-component host-guest RTP materials, two three-component room-temperature afterglow (RTA) materials (NA+RhB/ABDO and NA+RhB/DCB) also exhibited excellent thermal-responsive property (Fig. S33). This investigation might further expand the application scenarios of this strategy. The developed thermal-responsive design opens up an approach to the stimulus-responsive RTP materials. Predictably, some other factors that can increase the distance between the components of host-guest RTP systems would become the next stimuli of the new responsive RTP materials.

## 4. Methods

### 4.1. Preparation of the Host-Guest Doping RTP Materials

Host-guest doping RTP materials were prepared by a melt-casting method. Through a series of ratio optimization, the best emission ratio of each group of host-guest systems was selected for further preparation of host-guest doping RTP materials. The mass ratio of doping in each group is shown in Table S1.

### 4.2. Preparation of Host-Guest & Matrix Materials

80 mg NA/ABDO organic doping materials and 400 mg silica-OH were placed in a small quartz mortar; the host-guest doping RTP materials were grinded fully to be mixed with the silica-OH. Then, host-guest & silica-OH materials were filled in a quartz cuvette for further heating and spectral measurement process. Other systems were prepared in the same way, except that NA/DCB, NA/PCP, NA/TMBN, TPB/TPA, TPB/TPP, 2,3-NA/PCP, or other dispersion matrix were used to instead of NA/ABDO and normal-phase silica gel.

### 4.3. Heating and Spectral Measurement

The fluorescence spectra and phosphorescence spectra of NA/ABDO & silica-OH before heating were premeasured on a Hitachi F-7100 fluorospectrophotometer. And then, the quartz cuvette filled with NA/ABDO & silica-OH was placed in an oven at 70°C. After heating for 5 min, the quartz cuvette was taken out and placed in an ambient condition to cool to room temperature; then, the spectral information at 5 minutes of heating was measured. Then, put the quartz cuvette in the oven to heat another 5 minutes again, the spectrum was measured after cooling, and the spectrum information at this time was recorded as the data at the time of heating for 10 minutes. Repeated the above operation, heating time of 15, 20, 25, and 30 min was measured, respectively. Spectral data of each group of materials were measured under the same parameter conditions. The heating temperature, time, and excitation wavelength of each group of materials are shown in Table S1. The melting point of the used host and guest molecules is shown in Table S2.

### 4.4. Cycle Elution of NA/DCB

NA/DCB (1 : 800) dissolved in 5 mL dichloromethane was filled in a flask with 400 mg of silica-OH, spinning out of solvent and grinding to a mortar. The phosphorescence emission was quenched after heating for 90°C 30 min, and another 5 mL dichloromethane was filled in flask and sonicated for 5 min to elute NA/DCB from the silica gel. After respinning the solvent and regrinding, the phosphorescence emission was measured. Repeat the above operation for several times.

### 4.5. The Extraction and Recovery of TPB/TPA and NA/DCB

TPB/TPA (1 : 200) & silica-OH and NA/DCB (1 : 800) & silica-OH were firstly heating at 80°C 30 min and 90°C 30 min, respectively, to ensure the phosphorescence emission was quenched. Then, these two materials after heating were filled in separating funnel, followed by multiple extractions with dichloromethane. Water was added to promote the extraction efficiency. The collected eluent was spin-dried and remelted to obtain solid material. The phosphorescence spectra of TPB/TPA & silica-OH and NA/DCB & silica-OH were measured again.

### 4.6. ^1^H NMR

In order to maximize the extraction efficiency, TPB/TPA (1 : 50) and NA/DCB (1 : 50) were chosen to prepare the host-guest & silica-OH doping systems. TPB/TPA & silica-OH and NA/DCB & silica-OH were firstly heated at 80°C 30 min and 90°C 30 min, respectively, to ensure phosphorescence emission was quenched. Then, the hosts (TPA and DCB) and guests (TPB and NA) were, respectively, eluted by column chromatography from host-guest and silica-OH doping materials after heating. The eluent is as follows: TPB/TPA & silica-OH (dichloromethane) and NA/DCB & silica-OH (petroleum ether). The ^1^H NMR spectra of TPB, TPA, NA, and DCB were measured on a Bruker Advance DMX 400 spectrophotometer using CDCl_3_ as solvent.

### 4.7. HRMS

TPB/TPA (1 : 50) & silica-OH and NA/DCB (1 : 50) & silica-OH were firstly heated at 80°C 30 min and 90°C 30 min, respectively, to ensure phosphorescence emission was quenched. Then, host-guest & silica-OH doping materials after heating were filled in separating funnel followed by multiple extractions with dichloromethane. The collected eluent was spin-dried and re-melted to obtain solid material. Then, solid material was dissolved in dichloromethane for HRMS measurement. HRMS were recorded on a Bruker impact II10200 mass spectrometer.

### 4.8. Computational Details

All the calculated data are obtained using Gaussian 09 program package in this article. B3LYP method is used to fully optimize the geometries of species (TPB, TPA, NA, DCB, and silica-OH) based on DFT (density functional theory). C, O, N, and H elements are described by the double-*ζ* basis set (6-31G∗). The frequency calculations are calculated at the B3LYP/6-31G∗ level, verifying the rationality of all stationary points to get the thermal correction to Gibbs free energy (equilibrium structures: no imaginary frequency).

### 4.9. Dispersion of Fluorescent Dyes

Fluorescein & silica-OH (1 : 40) and rhodamine B & silica-OH (1 : 40) were firstly mixed by simply shaking, instead of grinding. Then, fluorescein & silica-OH was heated at 230°C for 30 min, and rhodamine B & silica-OH was heated at 180°C for 30 minutes. Fluorescein & NaCl (1 : 40) and rhodamine B & NaCl (1 : 40) were prepared and operated in the same way, except that NaCl was used instead of silica-OH.

## Figures and Tables

**Table 1:** Gibbs free energy change (*∆G*) of the two components in TPB/TPA & silica-OH systems.

Temperature (K)	*∆G* (kJ/mol)				
	TPB-TPB	TPB-TPA	TPA-TPA	TPB-silica-OH	TPA-silica-OH
298.15	44.2	38.6	35.8	22.3	26.1
393.15	59.2	50.9	46.7	43.4	41.4

**Table 2:** Gibbs free energy change (*∆G*) of the two components in NA/DCB & silica-OH systems.

Temperature (K)	*∆G* (kJ/mol)				
	NA-NA	NA-DCB	DCB-DCB	NA-silica-OH	DCB-silica-OH
298.15	13.5	13.0	16.5	-6.8	4.1
403.15	24.9	24.9	27.1	12.4	23.1

## Data Availability

All relevant data that support the findings are available within this article and supporting information and are also available from authors upon reasonable request.

## Supplementary Materials

Supplementary Materials Table S1: preparation conditions of different host-guest & matrix doping systems. Table S2: predicted and actually measured melting points of guests, hosts, and host-guest systems. Figure S1: the chemical structural formulas of the guests and hosts of doping systems. Photos of the host-guest doping RTP materials and host-guest & silica-OH materials, before and after heating for 30 minutes at a certain temperature under UV light irradiation and after the UV irradiation. Figure S2: trends of relative RTP intensity of host-guest doping RTP systems after heating for 5-30 minutes at a certain temperature. Figure S3: photos of the host-guest & silica-OR materials before and after heating for 30 minutes at a certain temperature under UV light irradiation and after the UV irradiation. Figure S4: effect investigation of the matrix on the thermal response. Figure S5: thermal response of TPB/TPA & silica-OH (80°C) and NA/DCB & silica-OH (90°C) with different mass ratios in 30 min. Figure S6: thermal response of TPB/TPA & silica-OR (80°C) with different mass ratios (1 : 1, 1 : 5, and 1 : 10) in 30 min. Figure S7: thermal response of TPB/TPA & silica-OH (1 : 5, 80°C) and NA/DCB & silica-OH (1 : 5, 90°C) in different mesh sizes (60-100, 300-400, and 500-800) in 30 min. Figure S8: trends of relative phosphorescence intensity of host-guest, host-guest & silica-OR, and host-guest & silica-OH materials after heating for 30 minutes at a certain temperature. Figure S9-S12: trends of relative phosphorescence intensity of TPB/TPA & silica-OR and TPB/TPA & silica-OH materials, 2,3-NA/PCP & silica-OR and 2,3-NA/PCP & silica-OH materials, NA/DCB & silica-OR and NA/DCB & silica-OH materials, and NA/ABDO & silica-OR and NA/ABDO & silica-OH materials after heating for 30 minutes at different temperatures. Figure S13-S16: trends of relative fluorescence (FL) intensity of TPB/TPA systems, 2,3-NA/PCP systems, NA/DCB systems, and NA/ABDO systems after heating for 30 minutes at different temperatures. Figure S17: trends of phosphorescence intensity of doping materials directly heating for 30 minutes at different temperatures. Figure S18: the phosphorescence lifetime of NA/DCB at 543 nm before and after heating at 90°C 30 min and NA/DCB & silica-OH before and after heating at 90°C 10, 20 min. *λ*ex =365 nm. Figure S19: recoverability investigation of the RTP emission of the NA/DCB & silica-OH system in six cycles. Figure S20-S21: high-resolution mass spectrometry of the extracted molecules from TPB/TPA & silica-OH system and NA/DCB & silica-OH system. Figure S22: the FTIR spectral characteristics of the NA/DCB group and TPB/TPA group before and after thermal treatment for 30 minutes (NA/DCB 90°C and TPB/TPA 80°C). Figure S23: the XRD characteristics of the silica-OH and silica-OR before and after thermal treatment at 90°C for 30 minutes. Figure S24: the XRD characteristics of the NA/DCB group and TPB/TPA group before and after thermal treatment for 30 minutes (NA/DCB 90°C; TPB/TPA 80°C). Figure S25: the fluorescence spectra of the fluorescein and rhodamine B in NaCl matrix. Figure S26: the fluorescence and phosphorescence photos and phosphorescence spectra of TPB/TPA & NaCl and NA/DCB & NaCl systems before and after heating for 200°C 30 min. Figure S27: a schematic diagram of possible mechanism of the thermal-responsive property. Figure S28: single stimulus investigation of the yellow emissive (NA/DCB & silica-OH) and the green emissive (TPB/TPA & silica-OH) host-guest RTP systems. Figure S29: the information decryption application based on the different thermal-responsive temperature of the NA/ABDO and NA/DCB systems. Figure S30: the relative spectral properties of Py/PCP system. Abbreviations: Py: pyrene; PCP: pentachloropyridine. Figure S31: trends of relative phosphorescence intensity and RTP emission images of Py/PCP and Py/PCP & silica-OH before and after heating for 30 minutes at 70°C. Figure S32: trends of relative phosphorescence intensity over 168 hours at the freezing temperature (-20°C) and room temperature (25°C).
