# Supplementary material for: Molecular Thermal Motion Modulated Room-Temperature Phosphorescence for Multilevel Encryption
Source: Research (Wash D C). 2022 Jul 23;2022:9782713. doi: 10.34133/2022/9782713 (PMC9351586; doi:10.34133/2022/9782713)
Supplement: Supplementary Materials — Table S1: preparation conditions of different host-guest & matrix doping systems. Table S2: predicted and actually measured melting points of guests, hosts, and host-guest systems. Figure S1: the chemical structural formulas of the guests and hosts of doping systems. Photos of the host-guest doping RTP materials and host-guest & silica-OH materials, before and after heating for 30 minutes at a certain temperature under UV light irradiation and after the UV irradiation. Figure S2: trends of relative RTP intensity of host-guest doping RTP systems after heating for 5-30 minutes at a certain temperature. Figure S3: photos of the host-guest & silica-OR materials before and after heating for 30 minutes at a certain temperature under UV light irradiation and after the UV irradiation. Figure S4: effect investigation of the matrix on the thermal response. Figure S5: thermal response of TPB/TPA & silica-OH (80°C) and NA/DCB & silica-OH (90°C) with different mass ratios in 30 min. Figure S6: thermal response of TPB/TPA & silica-OR (80°C) with different mass ratios (1 : 1, 1 : 5, and 1 : 10) in 30 min. Figure S7: thermal response of TPB/TPA & silica-OH (1 : 5, 80°C) and NA/DCB & silica-OH (1 : 5, 90°C) in different mesh sizes (60-100, 300-400, and 500-800) in 30 min. Figure S8: trends of relative phosphorescence intensity of host-guest, host-guest & silica-OR, and host-guest & silica-OH materials after heating for 30 minutes at a certain temperature. Figure S9-S12: trends of relative phosphorescence intensity of TPB/TPA & silica-OR and TPB/TPA & silica-OH materials, 2,3-NA/PCP & silica-OR and 2,3-NA/PCP & silica-OH materials, NA/DCB & silica-OR and NA/DCB & silica-OH materials, and NA/ABDO & silica-OR and NA/ABDO & silica-OH materials after heating for 30 minutes at different temperatures. Figure S13-S16: trends of relative fluorescence (FL) intensity of TPB/TPA systems, 2,3-NA/PCP systems, NA/DCB systems, and NA/ABDO systems after heating for 30 minutes at different temperatur [file 9782713.f1.zip › Supporting Information.docx]

**Supplementary Information for**

**Molecular Thermal Motion Modulated Room-Temperature Phosphorescence for Multilevel Encryption**

Jiaqiang Zhao^1^, Guojuan Yan^1^, Wei Wang^1^, Shishi Shao^1^, Binfang Yuan^2*^, Yan Jie Li^1^, Xuepeng Zhang^3*^, Cheng Zhi Huang^1^, Peng Fei Gao^1*^

**1. Instruments, Materials, and Methods**

All fluorescence spectra and phosphorescence spectra were recorded on a Hitachi F-7100 fluorescence spectrophotometer. The phosphorescence lifetime were measured on an Edinburgh FLS 1000 fluorescence spectrophotometer. Doping materials were placed in an electric heating constant temperature blast drying oven (DHG-9030A) from Shanghai Qixin Scientific Instrument Co., Ltd. to heat. ^1^H NMR (400 MHz) spectra were recorded on a Bruker Advance DMX 400 spectrophotometer using Chloroform-d (CDCl_3_) as solvent. High resolution mass spectra (HRMS) were recorded on a Bruker impact II10200 mass spectrometer. Fourier transform infrared (FT-IR) measurements were made by a FTIR-8400S spectrophotometer from Shimadzu, Japan. X-ray diffraction (XRD) measurements were made by a X'Pert3 Powder10300 from PANalytical. Photographs were taken by a Huawei P30 camera.

All chemicals, solvents and reagents were commercially available and used directly without further purification.

**2. Supporting tables and figures**

**Table S1.** Preparation conditions of different host-guest&matrix doping systems.

| Materials | H:G ^[a]^ | D:M ^[b]^ | Temperature/^o^C ^[c]^ | Time/min ^[d]^ | PL *λ*_ex_ /nm ^[e]^ | FL *λ*_ex_ /nm ^[f]^ |
| --- | --- | --- | --- | --- | --- | --- |
| NA/ABDO | 1:200 | 1:5 | 70 | 30 | 365 | 365 |
| NA/TMBN | 1:100 | 1:5 | 70 | 30 | 365 | 365 |
| NA/PCP | 1:100 | 1:5 | 70 | 30 | 254 | 365 |
| NA/DCB | 1:800 | 1:5 | 90 | 30 | 365 | 365 |
| TPB/TPP | 1:100 | 1:5 | 70 | 30 | 365 | 365 |
| TPB/TPA | 1:200 | 1:5 | 80 | 30 | 365 | 365 |
| 2,3-NA/PCP | 1:400 | 1:5 | 70 | 30 | 254 | 254 |
| Py/PCP | 1:200 | 1:5 | 70 | 30 | 254 | - |

[a] The mass ratio of host (H) and guest (G) in a doping material. [b] The mass ratio of host-guest material (D) to matrix (M). [c] The point of temperature in detail of each system. [d] The heating time of each system. [e] The excitation wavelength (*λ*_ex_) of photos under UV off and *λ*_ex_ of the phosphorescence emission spectrums (PL) of each system. [f] The excitation wavelength of photos under UV on and *λ*_ex_ of the fluorescence emission spectrums (FL) of each system.

**Table S2.** Predicted and actually measured melting points of guests, hosts and host-guest systems.

| Components | Compound abbreviations and melting points | |
| --- | --- | --- |
| Guest | NA: 269^o^C | TPB: 230-234^o^C |
|  | 2,3-NA: 246^o^C | Py: 148^o^C |
| Host | ABDO: 80-82^o^C | TMBN: 91-94^o^C |
|  | PCP: 124^o^C | DCB: 137-139^o^C |
|  | TPA: 124-128^o^C | TPP: 79-81^o^C |


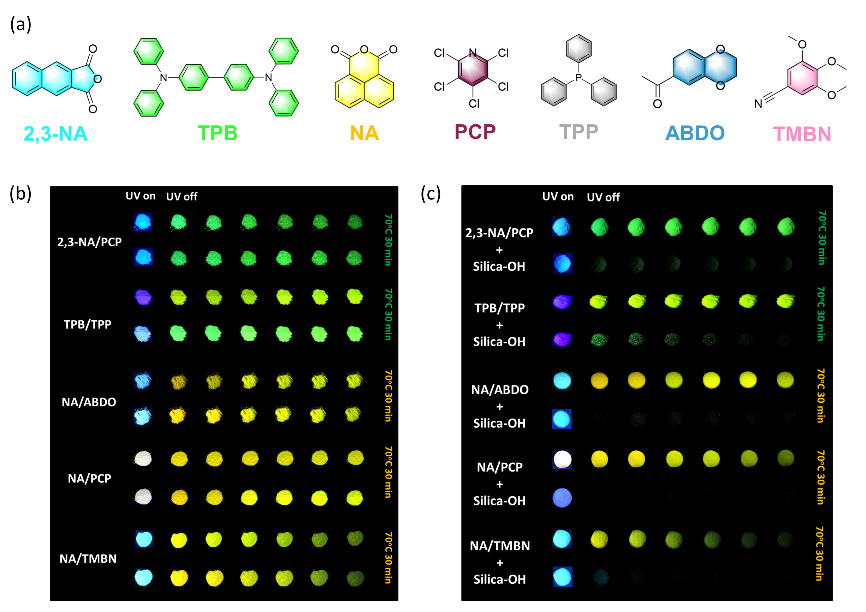


**Fig. S1** (a) The chemical structural formulas of the guests and hosts of doping systems, (b) Photos of the host-guest doping RTP materials before and after heating for 30 minutes at a certain temperature under UV light irradiation (left) and after the UV irradiation (right), (c) Photos of the host-guest&Silica-OH materials before and after heating for 30 minutes at a certain temperature under UV light irradiation (left) and after the UV irradiation (right).


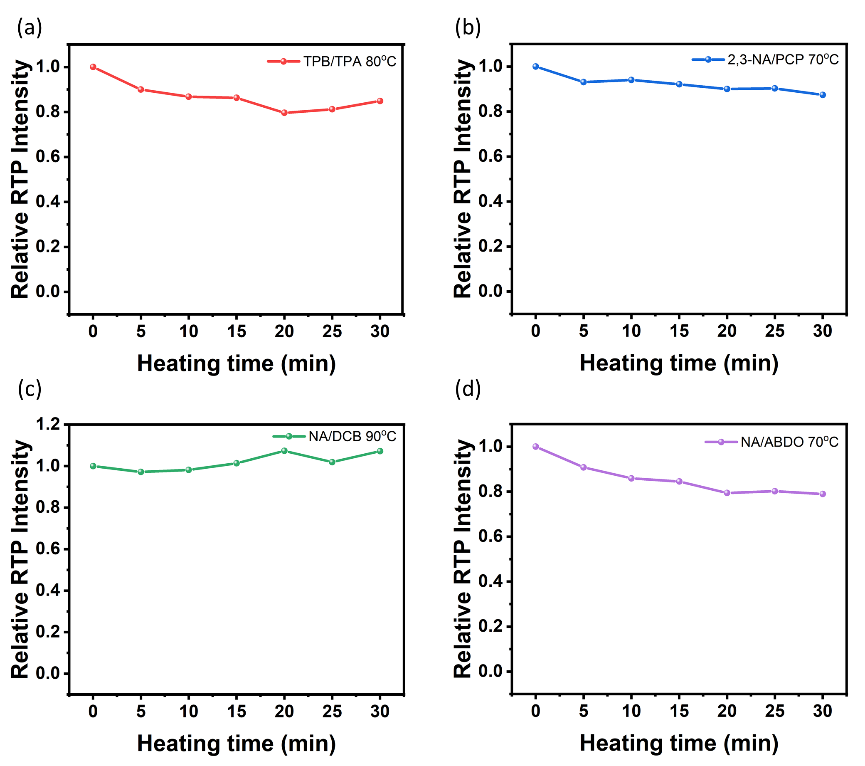


**Fig. S2** Trends of relative RTP intensity of host-guest doping RTP systems after heating for 5-30 minutes at a certain temperature. (a) TPB/TPA, (b) 2,3-NA/PCP, (c) NA/DCB, (d) NA/ABDO.


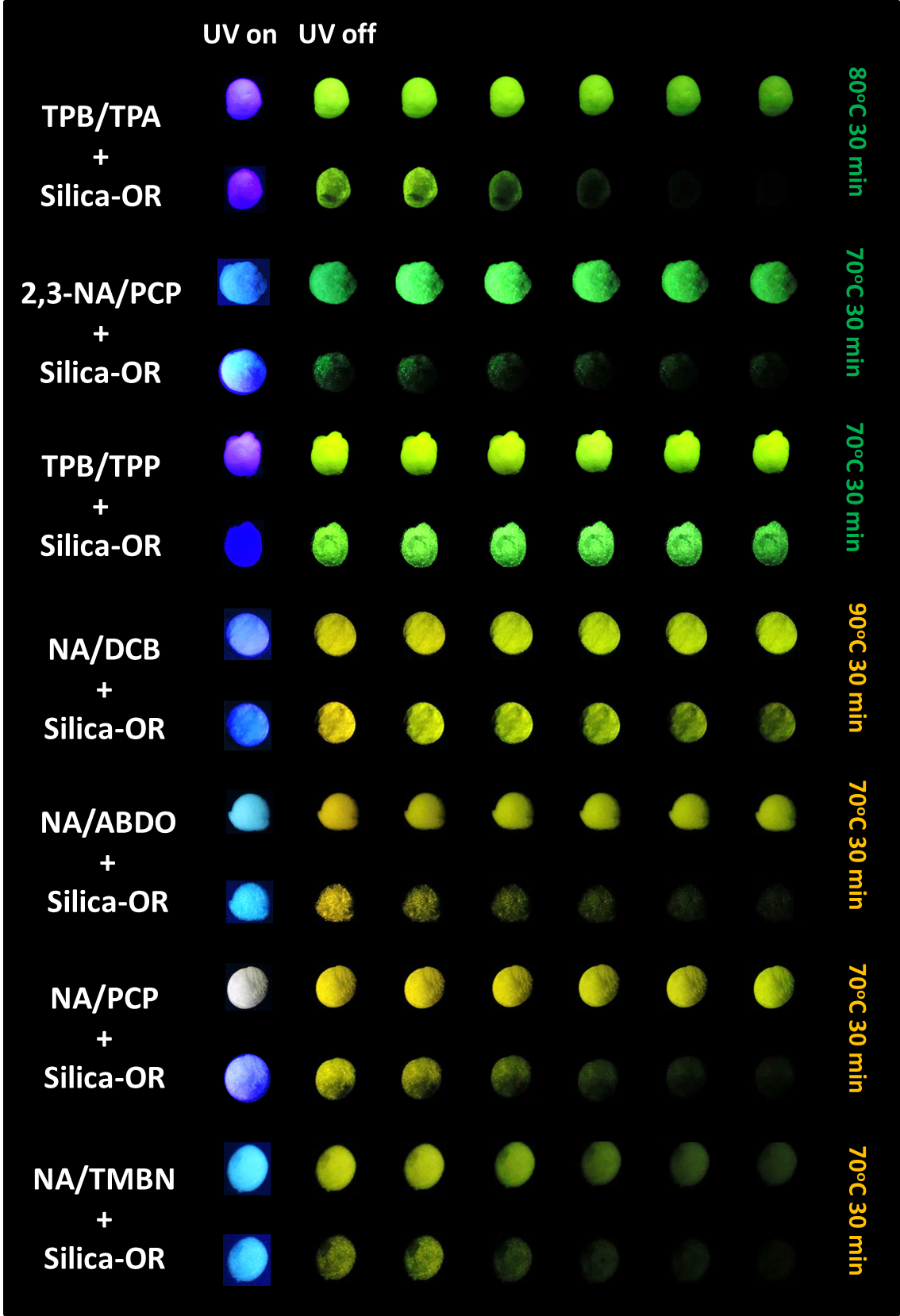


**Fig. S3** Photos of the host-guest&Silica-OR materials before (top) and after heating for 30 minutes (down) at a certain temperature under UV light irradiation (left) and after the UV irradiation (right).


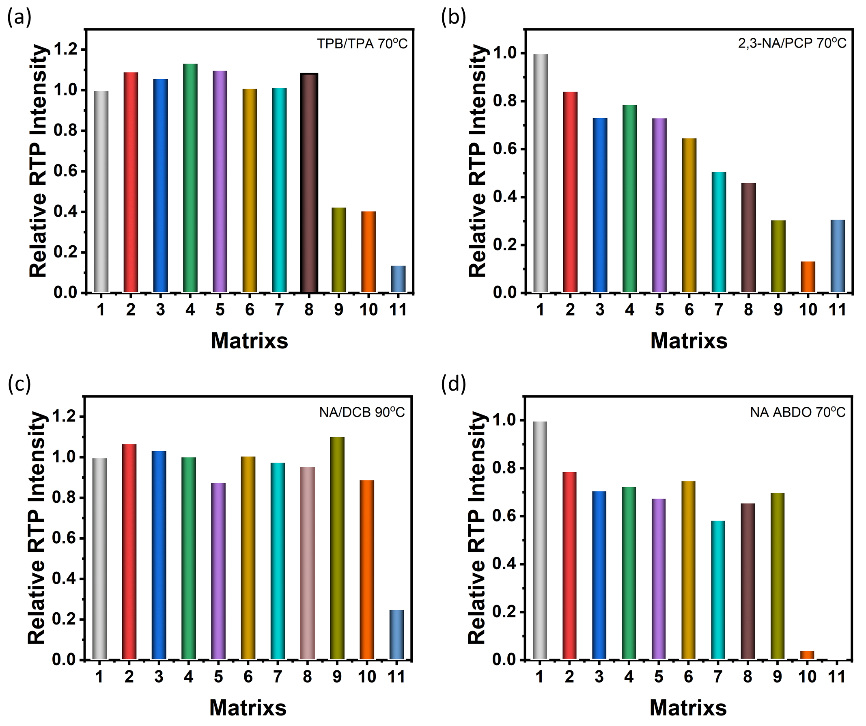


**Fig. S4** Effect investigation of the matrix on the thermal response. (a) The relative RTP intensity of TPB/TPA systems in different solid matrix, (b) 2,3-NA/PCP systems, (c) NA/DCB systems, (d) NA/ABDO systems. 1: Control, 2: host-guest, 3: host-guest&NaCl, 4: host-guest&KCl, 5: host-guest&Na_2_SO_4_, 6: host-guest&Sugar, 7: host-guest&Starch, 8: host-guest&Flour, 9: host-guest&Al_2_O_3_, 10: host-guest&Silica-OR, 11: host-guest&Silica-OH. The control means the initial phosphorescence intensity of the each group was normalized to “1”. Mass ratio between host-guest and matrix was 1:5.


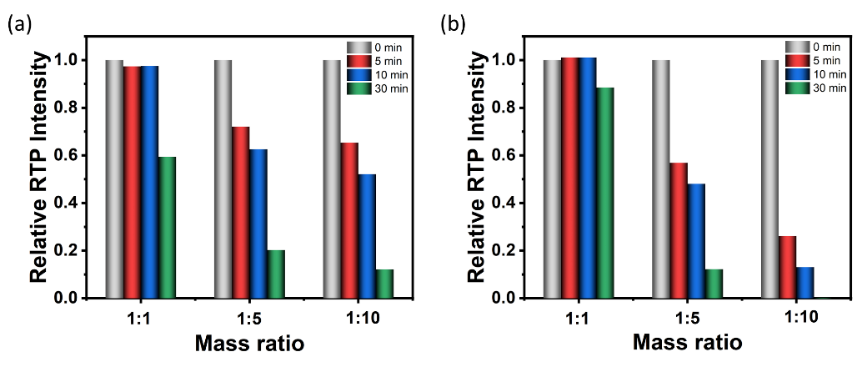


**Fig. S5** (a) Thermal response of TPB/TPA&Silica-OH (80^o^C) with different mass ratios (TPB/TPA:Silica-OH, 1:1, 1:5, 1:10) in 30 min, (b) Thermal response of NA/DCB&Silica-OH (90^o^C) with different mass ratios (1:1, 1:5, 1:10) in 30 min.


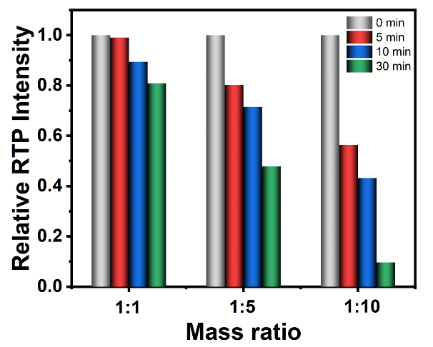


**Fig. S6** Thermal response of TPB/TPA&Silica-OR (80^o^C) with different mass ratios (1:1, 1:5, 1:10) in 30 min.


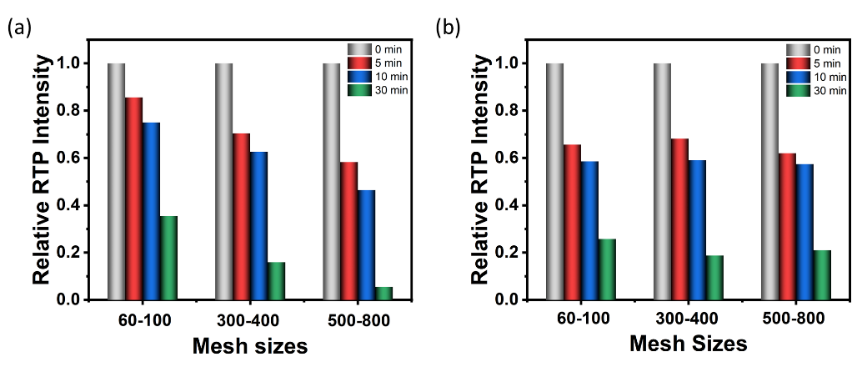


**Fig. S7** (a) Thermal response of TPB/TPA&Silica-OH (1:5, 80^o^C) in different mesh sizes (60-100, 300-400, 500-800) in 30 min, (b) Thermal response of NA/DCB&Silica-OH (1:5, 90^o^C) in different mesh sizes (60-100, 300-400, 500-800) in 30 min.


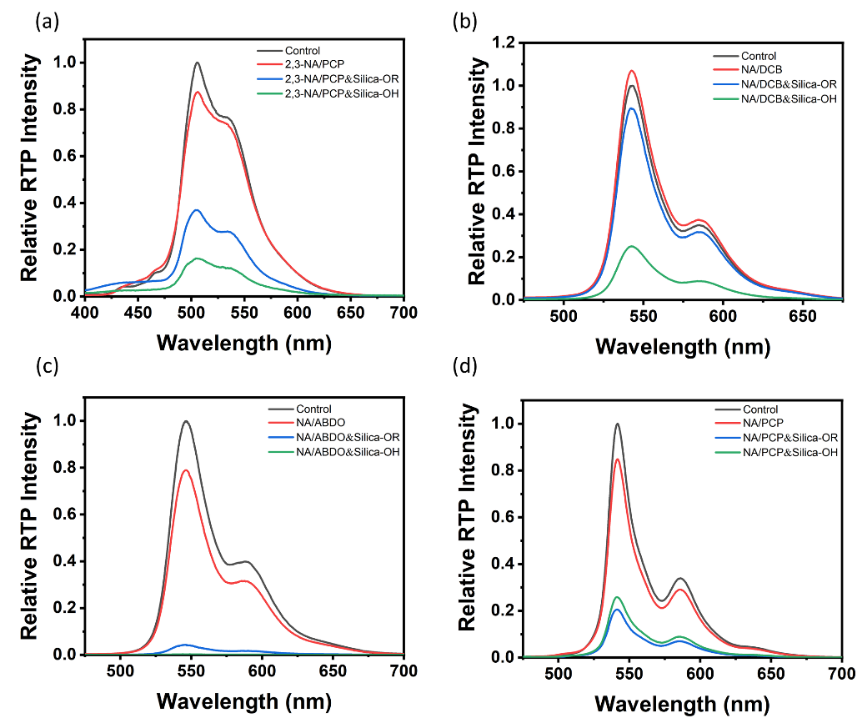


**Fig. S8** Trends of relative phosphorescence intensity of host-guest, host-guest&Silica-OR and host-guest&Silica-OH materials after heating for 30 minutes at a certain temperature. (a) 2,3-NA/PCP (70^o^C), (b) NA/DCB (90^o^C), (c) NA/ABDO (70^o^C), (d) NA/PCP (70^o^C). Control: The initial normalized phosphorescence intensity of host-guest, host-guest&Silica-OR and host-guest&Silica-OH materials.


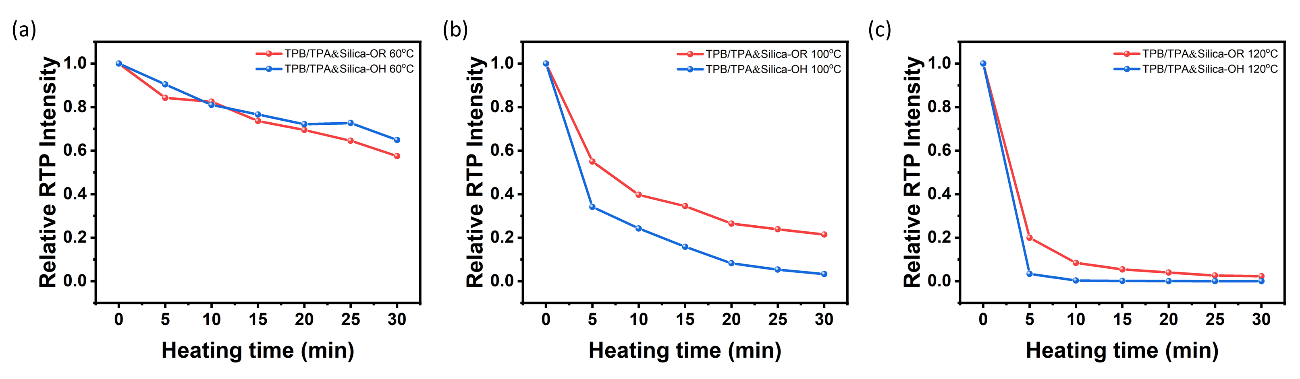


**Fig. S9** Trends of relative phosphorescence intensity of TPB/TPA&Silica-OR and TPB/TPA&Silica-OH materials after heating for 30 minutes at different temperatures. (a) 60^o^C, (b) 100^o^C, (c) 120^o^C.


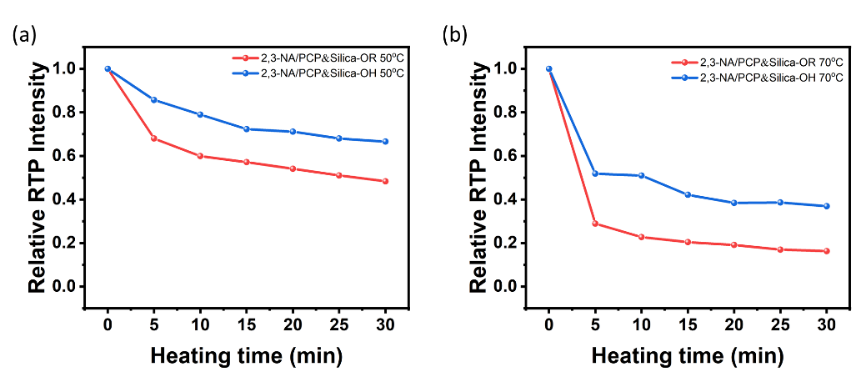


**Fig. S10** Trends of relative phosphorescence intensity of 2,3-NA/PCP&Silica-OR and 2,3-NA/PCP&Silica-OH materials after heating for 30 minutes at different temperatures. (a) 50^o^C, (b) 70^o^C.


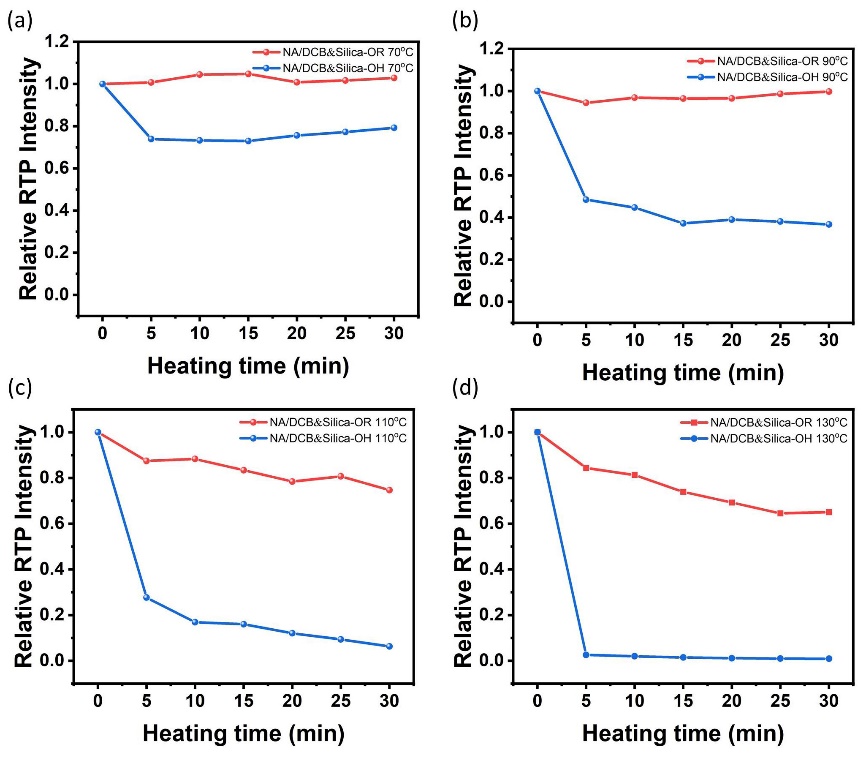


**Fig. S11** Trends of relative phosphorescence intensity of NA/DCB&Silica-OR and NA/DCB&Silica-OH materials after heating for 30 minutes at different temperatures. (a) 70^o^C, (b) 90^o^C, (c) 110^o^C, (d) 130^o^C.


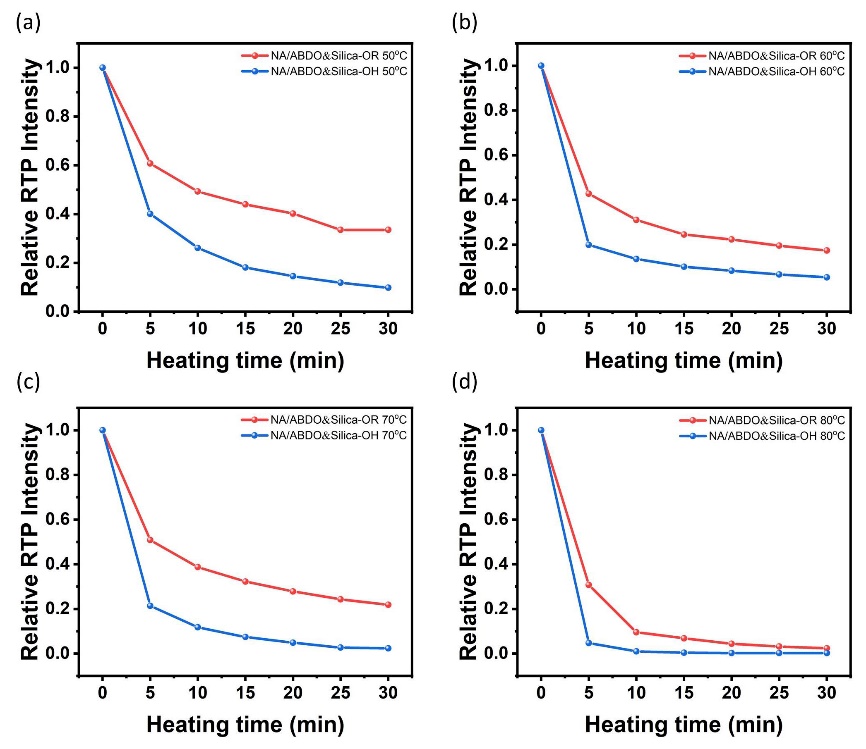


**Fig. S12** Trends of relative phosphorescence intensity of NA/ABDO&Silica-OR and NA/ABDO&Silica-OH materials after heating for 30 minutes at different temperatures. (a) 50^o^C, (b) 60^o^C, (c) 70^o^C, (d) 80^o^C.


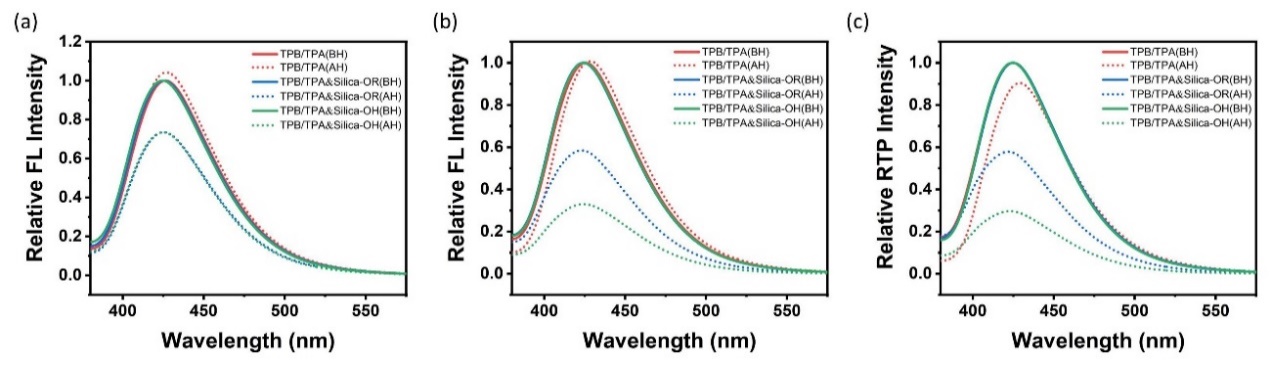


**Fig. S13** Trends of relative fluorescence (FL) intensity of TPB/TPA systems after heating for 30 minutes at different temperatures. (a) 60^o^C, (b) 100^o^C, (c) 120^o^C. Abbreviations: BH for before heating, AH for after heating.


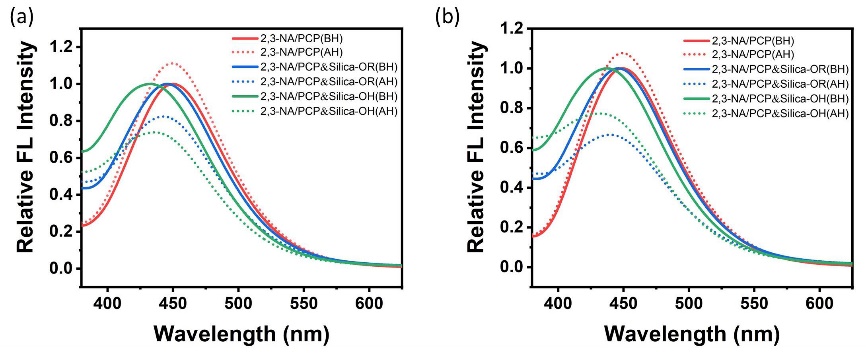


**Fig. S14** Trends of relative fluorescence intensity of 2,3-NA/PCP systems after heating for 30 minutes at different temperatures. (a) 50^o^C, (b) 70^o^C.


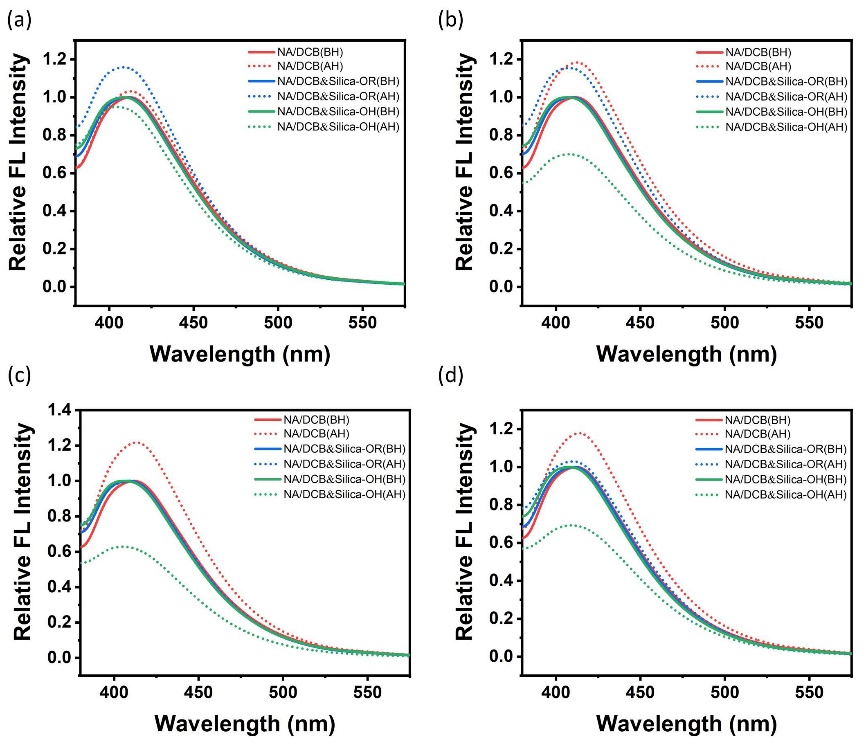


**Fig. S15** Trends of relative fluorescence intensity of NA/DCB systems after heating for 30 minutes at different temperatures. (a) 70^o^C, (b) 90^o^C, (c) 110^o^C, (b) 130^o^C.


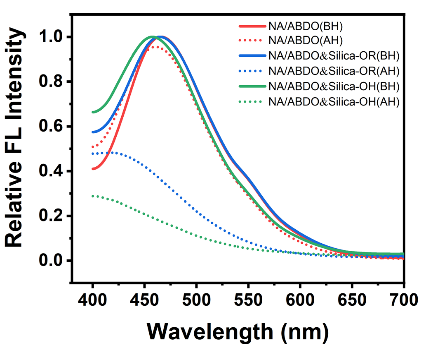


**Fig. S16** Trends of relative fluorescence intensity of NA/ABDO systems after heating for 30 minutes at 70^o^C.


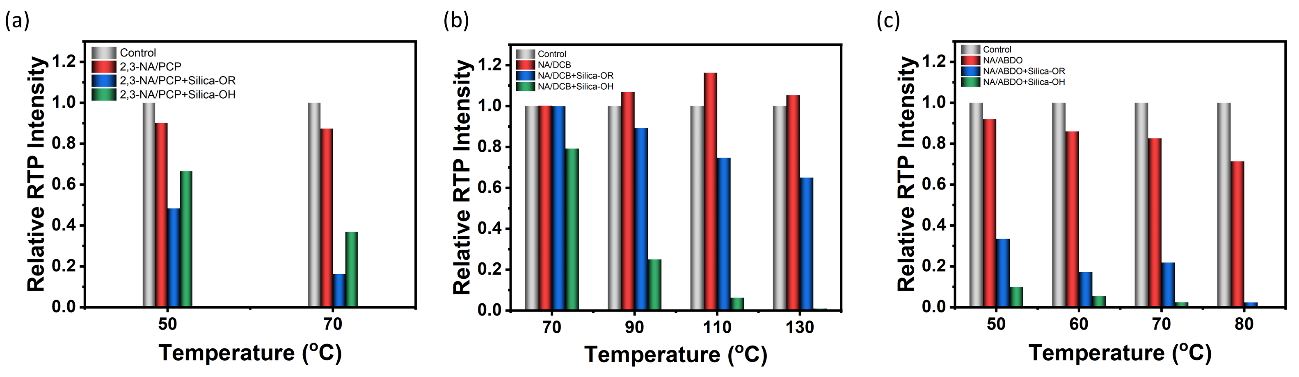


**Fig. S17** Trends of phosphorescence intensity of doping materials directly heating for 30 minutes at different temperatures. (a) 2,3-NA/PCP and related systems, (b) NA/DCB and related systems, (c) NA/ABDO and related systems. The control means the initial phosphorescence intensity of the each group was normalized to “1”.


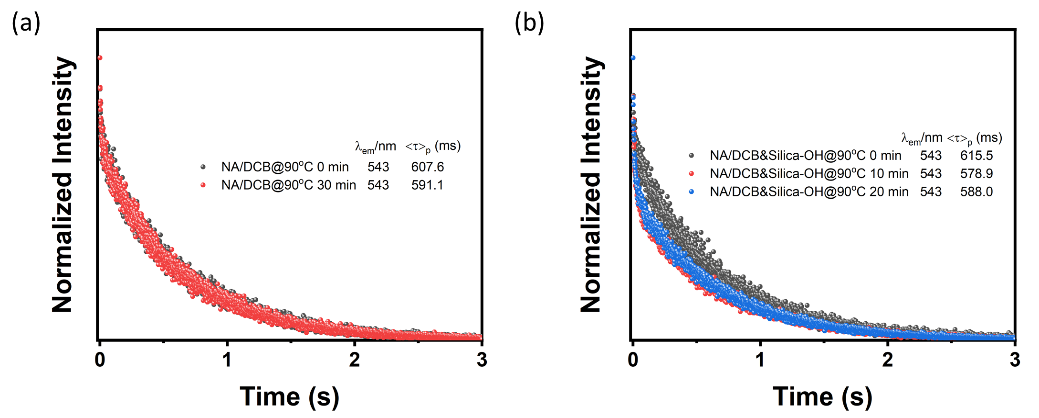


**Fig. S18** (a) The phosphorescence lifetime of NA/DCB at 543 nm before and after heating at 90^o^C 30 min. (b) The phosphorescence lifetime of NA/DCB&Silica-OH at 543 nm before and after heating at 90^o^C 10, 20 min. *λ*_ex_=365nm.


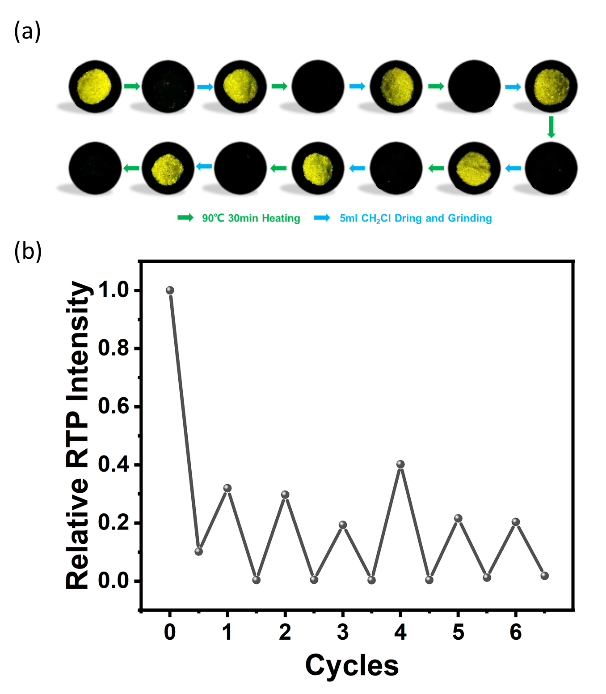


**Fig. S19** Recoverability investigation of the RTP emission of the NA/DCB&Silica-OH system in six cycles. (a) Photos of phosphorescence emission. (b) The corresponding relative phosphorescence intensities of NA/DCB&Silica-OH during heating and recovering.


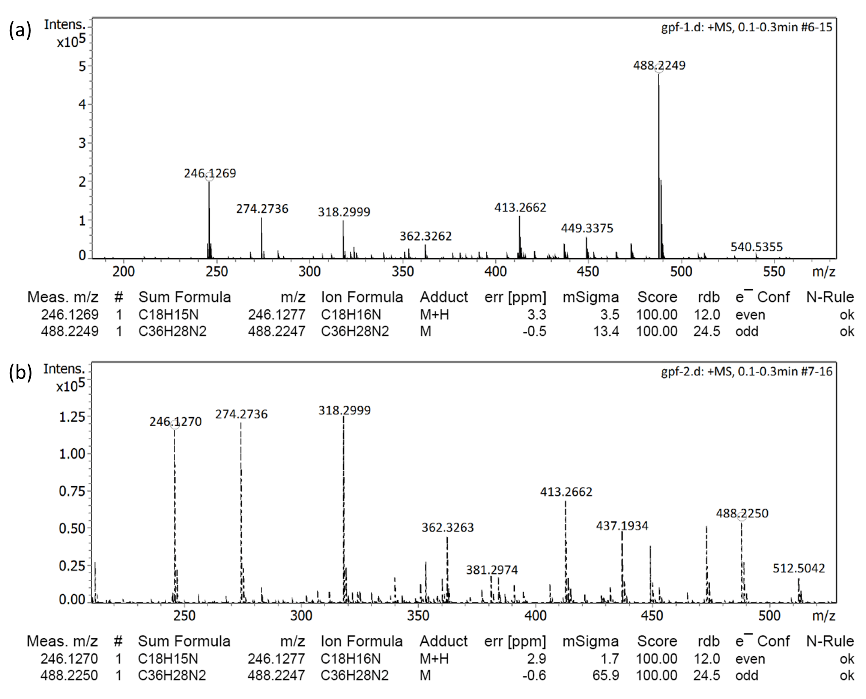


**Fig. S20** High-resolution mass spectrometry of the extracted molecules from TPB/TPA&Silica-OH system. (a) The directly doped TPB/TPA system without further operations. (b) The exracted materials from TPB/TPA&Silica-OH system after the thermal erasure of the RTP emission.


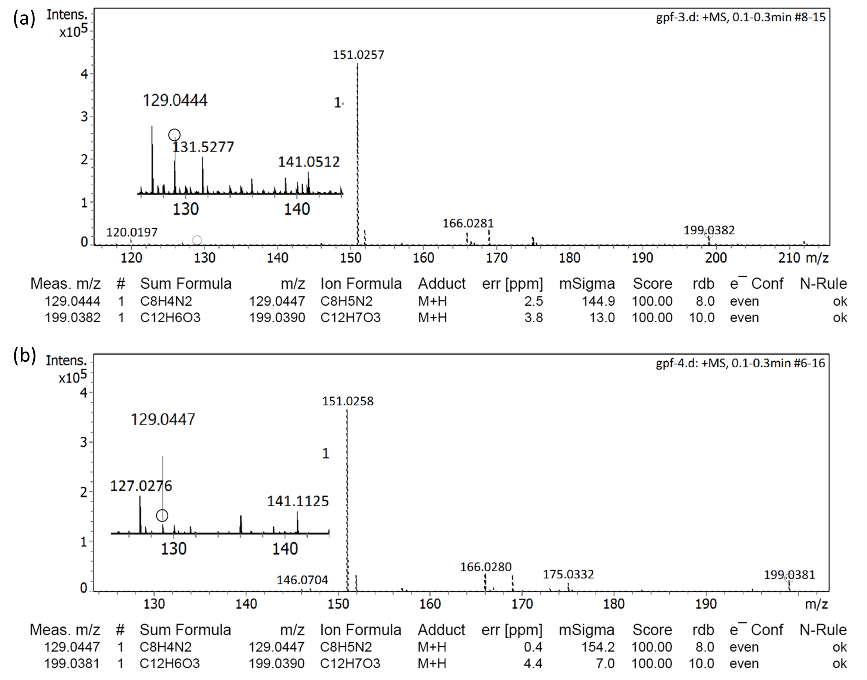


**Fig. S21** High-resolution mass spectrometry of the extracted molecules from NA/DCB&Silica-OH system. (a) The directly doped NA/DCB system without further operations. (b) The exracted materials from NA/DCB&Silica-OH system after the thermal erasure of the RTP emission. Insert: the enlarged area around m/z 129.

**
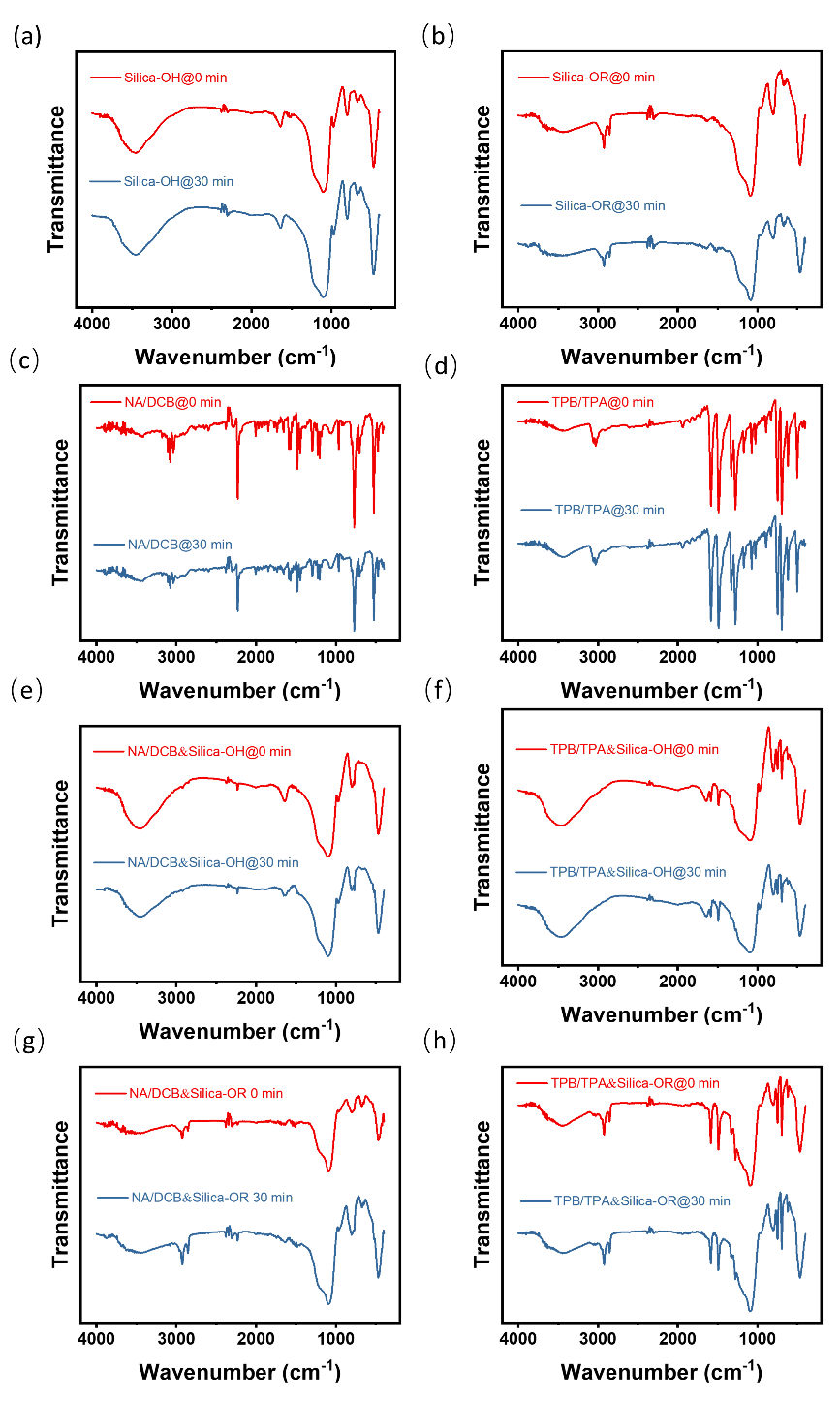
**

**Fig. S22** The FTIR spectral characteristics of the NA/DCB group and TPB/TPA group before and after thermal treatment for 30 minutes (NA/DCB 90^o^C; TPB/TPA 80^o^C). (a, b) The FTIR spectral characteristics of the Silica-OH and Silica-OR (90^o^C, 30 minutes). The FTIR spectral characteristics of the (c, e, g) NA/DCB group and (d, f, h) TPB/TPA group.

**
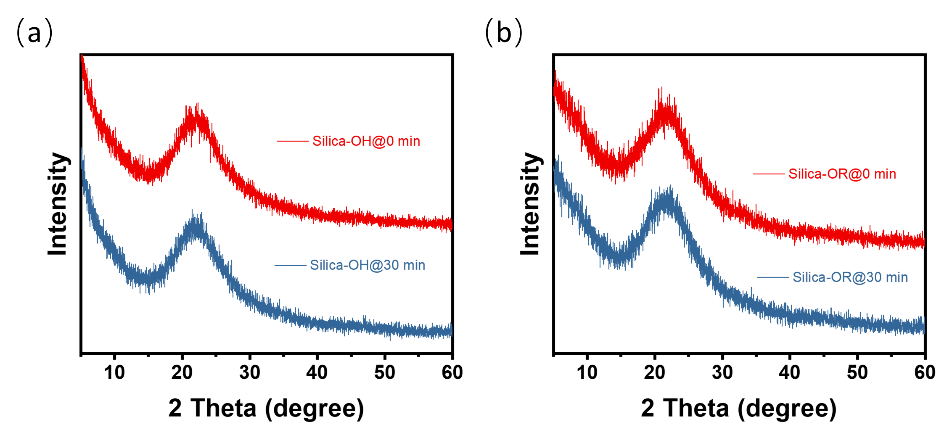
**

**Fig. S23** The XRD characteristics of the (a) Silica-OH and (b) Silica-OR before and after thermal treatment at 90^o^C for 30 minutes.

**
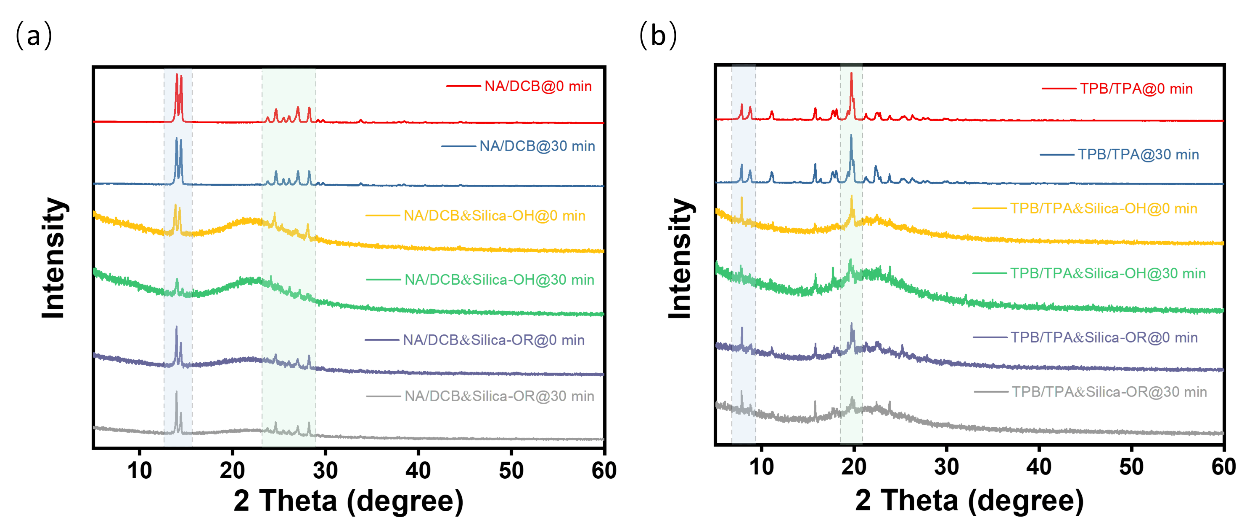
**

**Fig. S24** The XRD characteristics of the (a) NA/DCB group and (b) TPB/TPA group before and after thermal treatment for 30 minutes (NA/DCB 90^o^C; TPB/TPA 80^o^C).


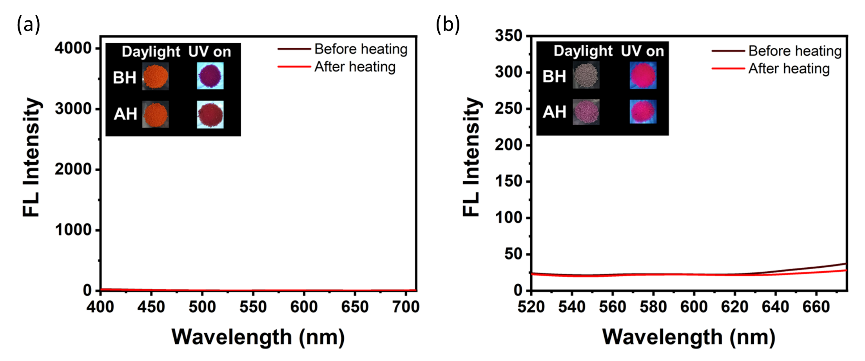


**Fig. S25** The fluorescence spectra of the Fluorescein and Rhodamine B in NaCl matrix. (a) The fluorescence intensity of Fluorescein&NaCl (1:40) before and after heating at 230^o^C for 30 minutes, (b) The fluorescence intensity of Rhodamine B&NaCl (1:40) before and after heating at 180^o^C for 30 minutes. Insert: the corresponding photos of the fluorescence emission.


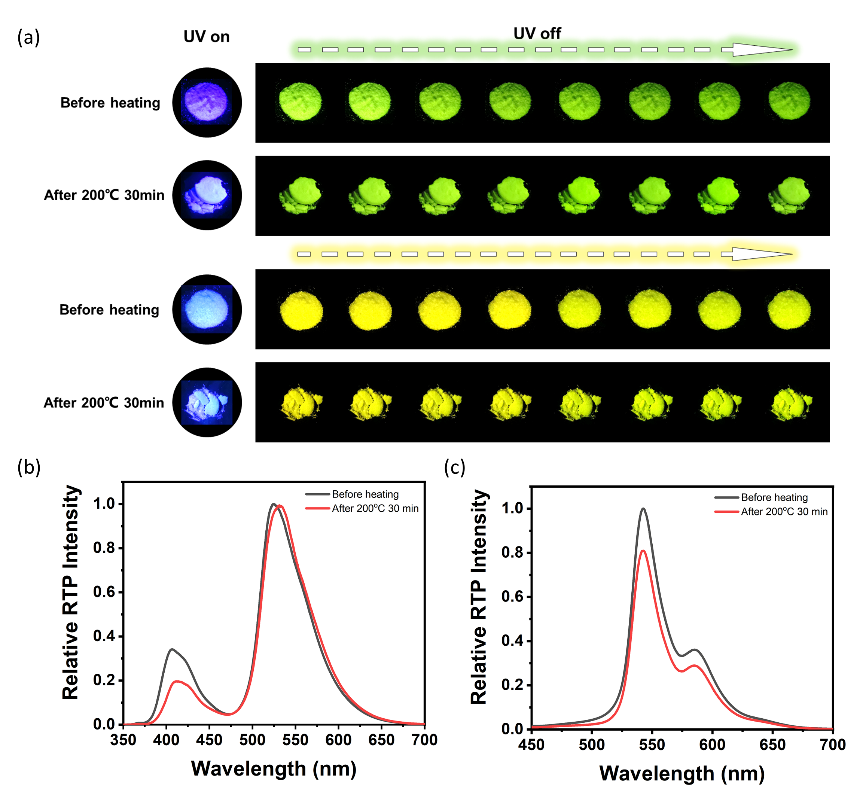


**Fig. S26** (a) The fluorescence and phosphorescence photos of host-guest&NaCl systems before and after heating for 200^o^C 30 min, the top one is TPB/TPA&NaCl, the bottom one is NA/DCB&NaCl, (b) phosphorescence spectra of TPB/TPA&NaCl before and after heating for 200^o^C 30min, (c) phosphorescence spectra of NA/DCB&NaCl before and after heating for 200^o^C 30min.


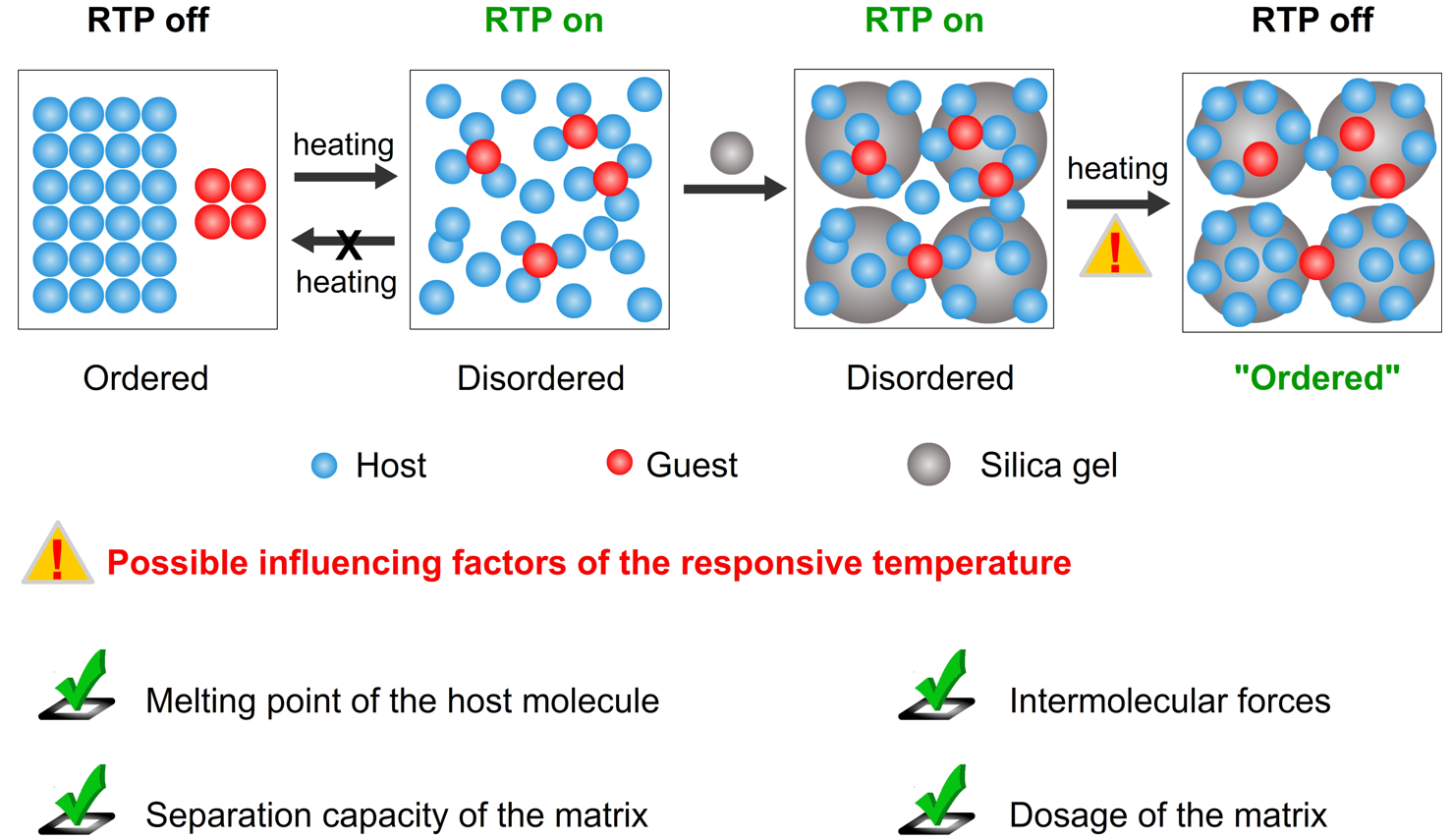


**Fig. S27** A schematic diagram of possible mechanism of the thermal-responsive property.


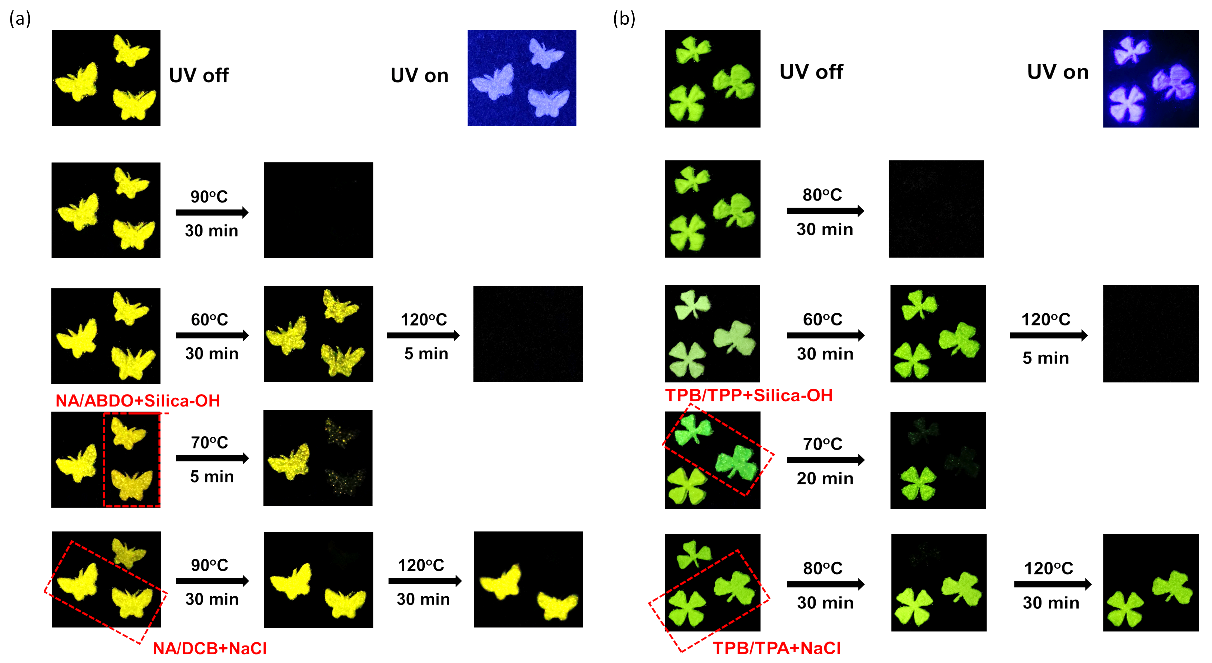


**Fig. S28** Single stimulus investigation of the (a) yellow emissive and the (b) green emissive host-guest RTP systems. Besides the noted NA/ABDO in (a) and TPB/TPP in (b), all the yellow emissive systems were NA/DCB&Silica-OH systems, and all the green emissive systems were TPB/TPA&Silica-OH systems.


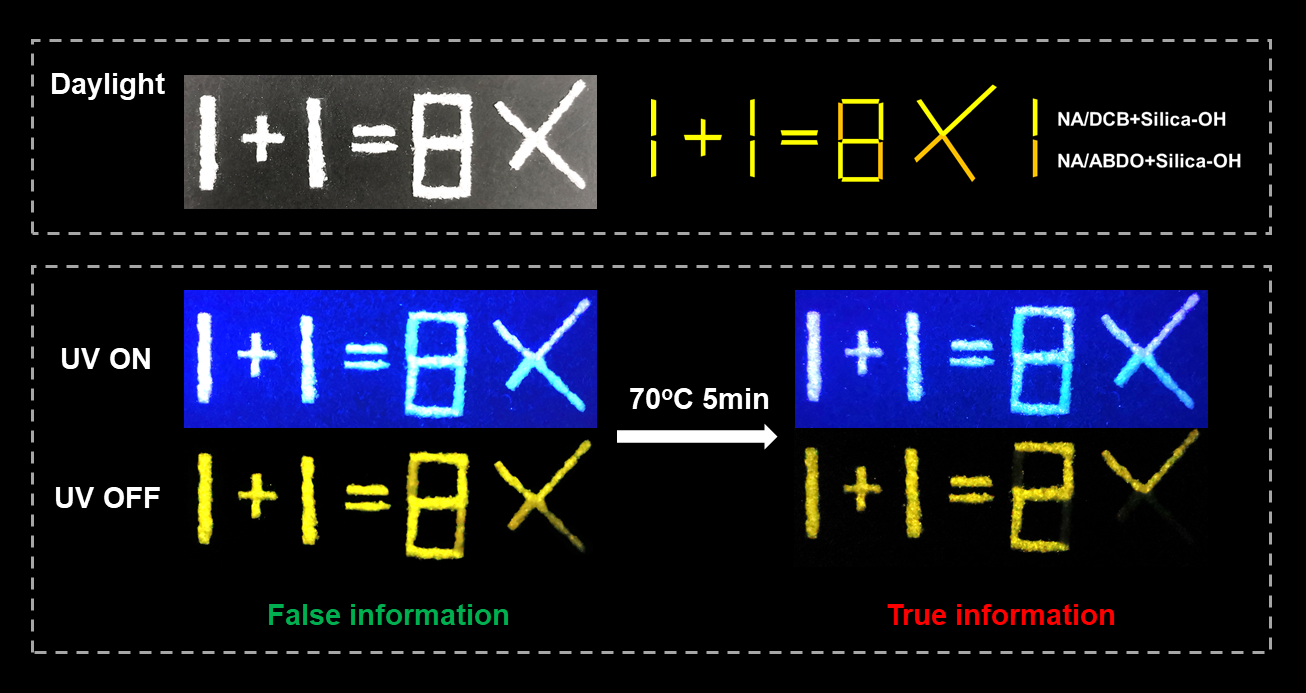


**Fig. S29** The information decryption application based on the different thermal-response temperature of the NA/ABDO and NA/DCB systems.


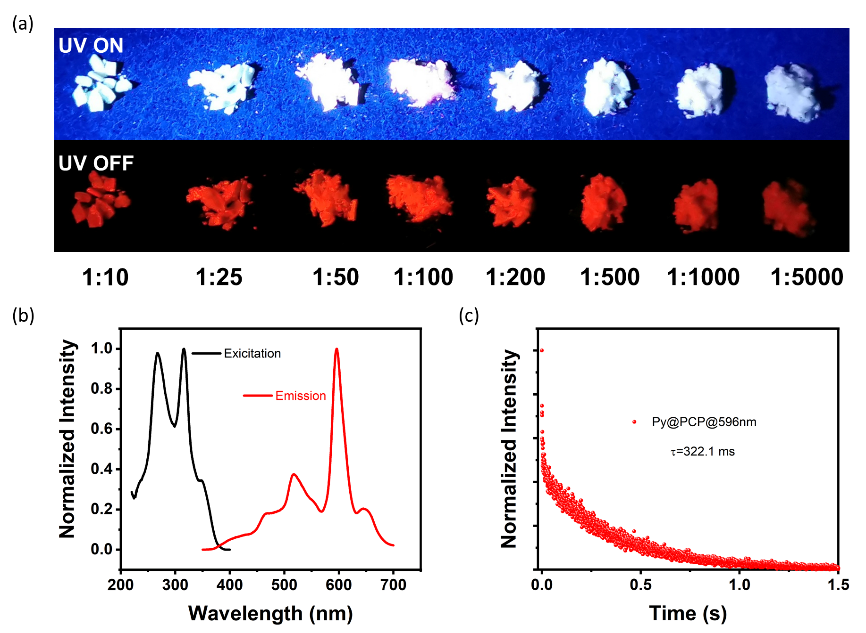


**Fig. S30** (a) The fluorescence (top) and phosphorescence (down) photos of the Py/PCP with different amount of Py (Mass ratio). (b) The phosphorescence excitation and emission spectra of Py/PCP (1:200). (c) The phosphorescence lifetime of Py/PCP (1:200) at 596 nm, *λ*_ex_=254 nm, . Abbreviations: Py for Pyrene, PCP for Pentachloropyridine.


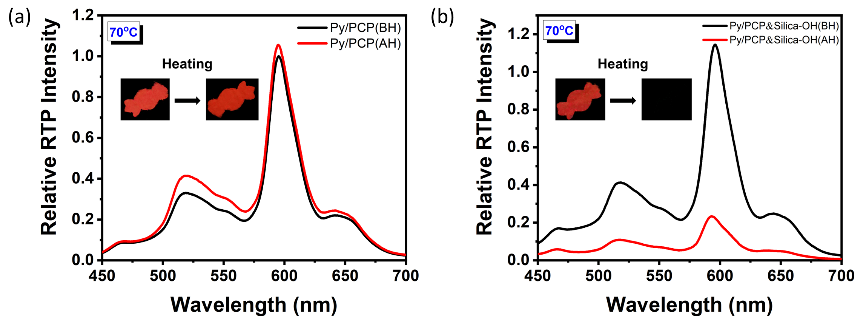


**Fig. S31** Trends of relative phosphorescence intensity and RTP emission images of Py/PCP (a) and Py/PCP&Silica-OH (b) before and after heating for 30 minutes at 70^o^C.


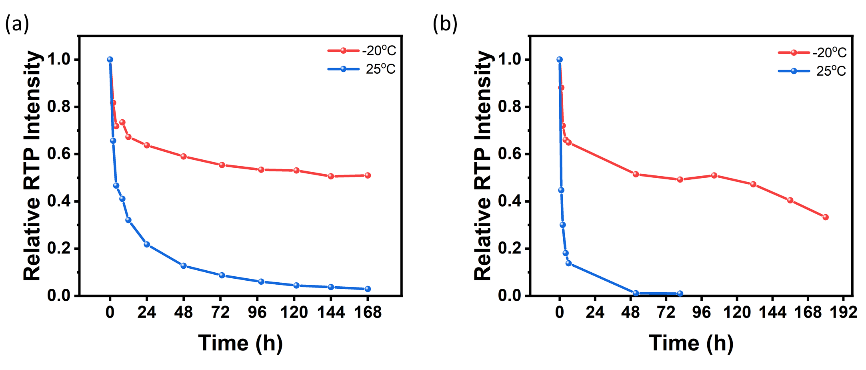


**Fig. S32** Trends of relative phosphorescence intensity over 168 hours at the freezing temperature (-20^o^C) and room temperature (25^o^C). (a) TPB/TPP&Silica-OH (1:5), (b) NA/ABDO&Silica-OH (1:5).


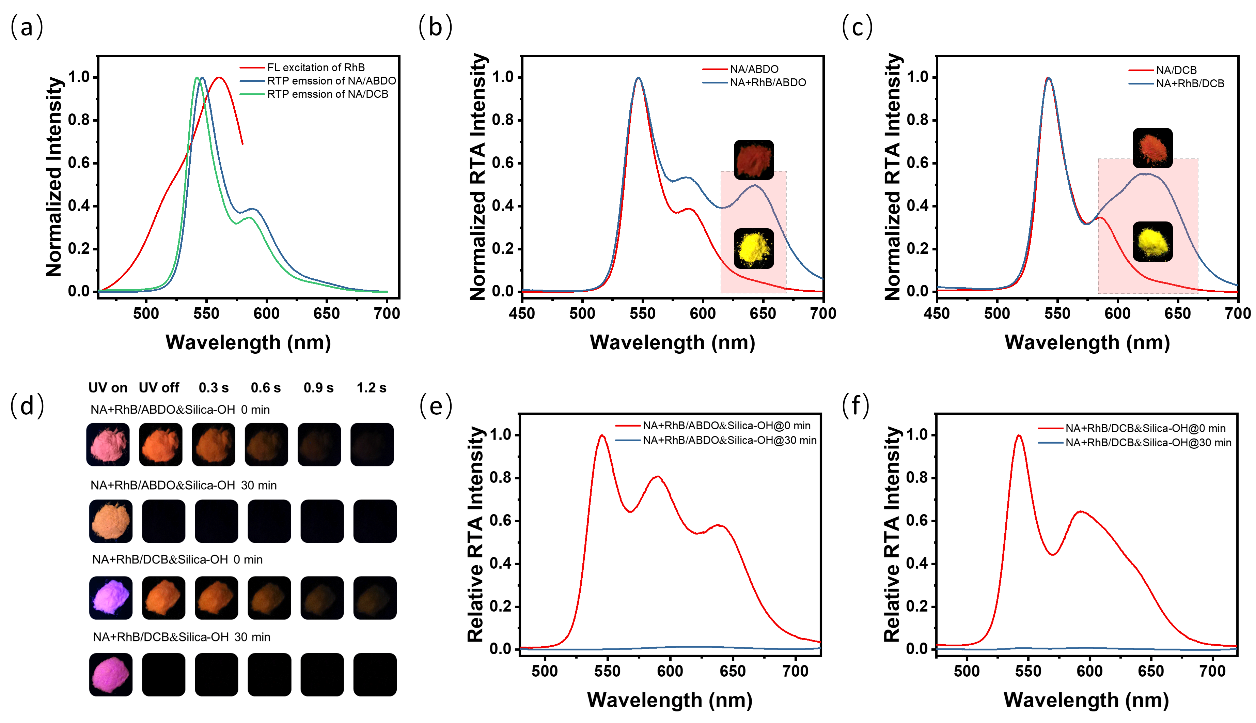


**Fig. S33** The investigation of the thermal-responsive property of three-component room-temperature afterglow (RTA) systems. (a) The phosphorescence spectra of the NA/ABDO and NA/DCB systems and the excitation spectra of RhB. The RTA emission spectra of (b) NA+RhB/ABDO and (c) NA+RhB/DCB. (d) The photos and (e, f) spectra of the thermal erasure of the RTA emission of NA+RhB/ABDO (Silica-OH, 70^o^C) and NA+RhB/DCB (Silica-OH, 90^o^C).
